# Mitigating Mobile‐Ion‐Induced Instabilities and Performance Losses in 2D Passivated Perovskite Solar Cells

**DOI:** 10.1002/adma.202501588

**Published:** 2025-05-09

**Authors:** Biruk Alebachew Seid, Sercan Ozen, Andrés‐Felipe Castro‐Méndez, Dieter Neher, Martin Stolterfoht, Felix Lang

**Affiliations:** ^1^ Physik und Optoelektronik weicher Materie Institut für Physik und Astronomie Universität Potsdam 14476 Potsdam‐Golm Germany; ^2^ Electronic Engineering Department The Chinese University of Hong Kong Hong Kong SAR 00000 China

**Keywords:** 2D passivation stability, ammonium salt passivation, mobile ion density, perovskite solar cells (PSCs), short‐circuit current density (*J*SC)

## Abstract

Bulky ammonium salt‐based passivation is an effective strategy for enhancing the performance and stability of perovskite solar cells (PSCs). Especially, phenethylammonium iodide (PEAI) is known to greatly improve open‐circuit voltage (*V*_OC_) and fill factor (FF). Despite these benefits, PEAI passivation leads to substantial short‐circuit current density (*J*_SC_) losses and rapid degradation under operational conditions. In this work, it is revealed that the *J*_SC_ loss as well as the accelerated degradation in PEAI‐passivated devices is caused by an increased mobile ion density. To mitigate this performance and stability‐limiting mechanism, ultrathin layers of ammonium benzenesulfonate (ABS) and/or ethylenediammonium diiodide (EDAI_2_) salts are then introduced between the PEAI and the perovskite, which stabilize the 2D perovskite layer and impede diffusion even under upon prolonged illumination. This leads to a reduced mobile ion density both in fresh devices and in the long term, lowering losses *J*_SC_, and thus enables power conversion efficiencies of ≈25% with enhanced stability. Overall, this study not only addresses the limitations of PEAI‐based 2D passivation but also paves the way for understanding 2D‐induced ionic *J*_SC_ losses.

## Graphical abstract

This study addresses the challenges of mobile‐ion‐induced instability in perovskite solar cells. Introducing bilayer passivation with ammonium salts (EDAI₂/PEAI and ABS/PEAI) significantly reduces ion density and improves device stability. The approach mitigates the detrimental effects of PEAI‐induced ionic losses, enhancing both performance and operational lifetime under continuous illumination.

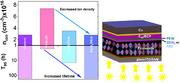

## 1 Introduction

Recent studies predict that perovskite single‐junction solar cells (PSCs) could reach power conversion efficiencies (PCEs) exceeding 30%, making them highly interesting for commercialization.^[^ ^1^, ^2^ ^]^ The main reason that PSCs have not yet reached this record efficiency are defects at the surface and grain boundaries of the perovskite, causing non‐radiative recombination of charge carriers.^[^ ^3^, ^4^ ^]^ A promising approach to passivate the perovskite interfaces is the application of bulky ammonium salts on the surface. These form a 2D perovskite phase that can reduce surface defects, suppressing non‐radiative recombination, and improve the energy‐alignment between the perovskite and C_60_, thereby enhance both the open‐circuit voltage (*V*_OC_) and fill factor (FF) of PSCs.^[^ ^5^, ^6^, ^7^, ^8^, ^9^, ^10^, ^11^ ^]^ To this end, various bulky ammonium salts such as phenethylammonium iodide (PEAI), n‐butylammonium bromide (n‐BABr), phenethylammonium chloride (PEACl), octylammonium iodide (OAI), cyclohexylethylammonium iodide (CEAI), ethylenediammonium diiodide (EDAI_2_), ethylenediammonium dichloride (MDACl_2_), among others, have been widely applied at the perovskite surface to construct 2D/3D heterostructures for both inverted p‐i‐n and n‐i‐p PSCs configurations.

PEAI has become a widely used agent for surface modification in PSCs among various passivating materials. It effectively forms a quasi‐2D and 2D perovskite structure that passivates the 3D perovskite interface, leading to enhanced overall device performance.^[^ ^12^, ^13^, ^14^, ^15^, ^16^, ^17^ ^]^ Moreover, the PEAI 2D interface passivation improves perovskite stability against moisture degradation due to its hydrophobic nature.^[^ ^18^, ^19^ ^]^ However, despite these advantages, recent studies have revealed that PEAI passivation leads to unwanted losses in *J*_SC_, which can negatively impact the device's efficiency.^[^ ^20^, ^21^ ^]^ In many reports, this *J*_SC_ loss is widely overlooked and has previously been reported to be caused by the formation of a low‐conductivity 2D passivation layer.^[^ ^15^, ^16^, ^17^, ^18^, ^19^, ^20^, ^21^ ^]^ Lower conductivity in the direction perpendicular to the (001) plane of the inorganic sheets is an issue in 2D‐passivated perovskite cells.^[^ ^15^, ^16^, ^17^, ^18^, ^19^, ^20^, ^21^ ^]^ However, despite the lower conductivity in this direction, PEAI passivation has demonstrated a significant increase in the FF, which contradicts the argument of a lower conductivity being the dominant limiting factor. For example, Lucarelli et al. employed layers of PEAI at different molar concentrations on the 3D flexible perovskite surface, achieving an improvement in *V*_OC_ and FF by increasing the concentration to 20 mm while the *J*_SC_ drops in opposite trend even at optimum concentration.^[^ ^13^ ^]^ In another study, Chen et al. utilized PEAI in MAPbI_3_‐based PSC devices, demonstrating that, despite the incorporation of a small amount of PEA_2_PbI_4_, the devices still exhibited a lower *J*_SC_ compared to those based solely on MAPbI_3_.^[^ ^22^ ^]^ A similar trend was also observed in another report by Chen et al, incorporating PEABr to form a 2D/3D device, with high *V*_oc_ and FF.^[^ ^23^ ^]^ To highlight the universal challenges associated with *J*_SC_ ​losses in 2D‐passivated PSCs, Figure S1 (Supporting Information) illustrates the current loss versus *V*_OC_ ​ improvement observed in previous studies from literature.

In addition to this *J*_SC_ losses more and more reports find lower operational stability.^[^ ^24^, ^25^, ^26^ ^]^ For instance, Park et al. applied PEAI passivation on top of triple‐cation PSCs and investigated the stability of the devices at 85 °C and 50% RH with MPP tracking.^[^ ^27^ ^]^ They found that PEAI‐based PSCs have limited operational thermal stability, at 65 °C over 500 h. Furthermore, they report the decomposition of PEAI‐passivated perovskite surfaces into PbI_2_ after just 2 h of thermal aging at 85 °C. They attributed this process to the diffusion of PEA cations into the bulk perovskite under thermal stress.^[^ ^27^ ^]^ Another study by Perini et al. investigated PEAI‐passivated perovskite films along with other bulky cation‐based films and demonstrated that the PEAI passivation molecules deposited at the interface slowly permeate into the perovskite film, especially under thermal stress (1 sun illumination at 55 °C in an inert atmosphere). This phenomenon led to a reconstruction of the crystalline structure at the interface that was correlated with the performance reduction.^[^ ^26^ ^]^ However, despite these observations, the underlying mechanisms responsible for *J*_SC_ loss and instabilities in these studies remain largely unexplored, and no systematic mitigation strategies have been proposed. This study therefore delves into the root causes of *J*_SC_ loss associated with PEAI passivation and introduces a novel approach to mitigate this challenge.

In this study, we utilize fast hysteresis (FH) *J–V*, bias‐assisted charge extraction (BACE), and photoluminescence (PL) techniques to investigate the impact of PEAI passivation on the *J*_SC_ loss and stability in PSCs. Our findings reveal that PEAI passivation increases the concentration of mobile ions, resulting in an average *J*_SC_ loss of 1.3 mA cm^−2^ due to screening of the field by the accumulation of ions at the interfaces. Moreover, we find that the accelerated degradation under operation (1 sun, 40 °C) is primarily caused by increased ionic *J*_SC_ losses of 14 mA cm^−2^ after 160 h of stabilized power output (SPO) tracking, while the ion‐freeze *J*_SC_ and performance are unaffected. The rapid degradation of devices passivated solely with PEAI is hence linked with an increased density of mobile ions. To address this, we then introduced additional interlayers using ammonium benzoate (ABS), or ethylenediammonium iodide (EDAI_2_). These interlayers reduced the initial mobile ion density and their evolution over time, lowered ionic losses, and increased the energy‐lifetime yield by enhancing operational stability of PEAI‐passivated devices.

## 2 Results and Discussion

### 2.1 PEAI Passivation and *J*_SC_ Losses

**Figure 1:**
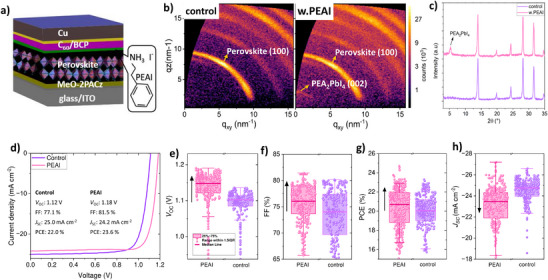
Comparisons of photovoltaic parameters of control and 2D/3D PSCs. a) Device schematic showing the 2D/3D inverted triple cation triple halide perovskites investigated in this work. PEAI was spin‐coated on top of perovskite. b) GIWAXS images of control, and PEAI‐passivated devices taken at an incident angle of 3 °c) The corresponding XRD pattern as a function incident angle. d) *JV* curves at a scan rate of 20 mV s^−1^ under AM1.5G illumination of champion devices and the corresponding parameters as insets. e,f,g,h) Statistics of comparison of PV parameters of control and PEAI‐passivated devices taken from 75 individual solar cells and active area of 6 mm^2^: e) open circuit voltage (*V*oc), f) fill factor (FF), g) power conversion efficiency (PCE), and h) short circuit current density (*J*_SC_).

To investigate the role of PEAI we first optimized the PEAI‐passivation for our p‐i‐n‐based devices utilizing a triple cation triple halide perovskite with the composition of Cs_0.05_(MA_0.05_FA_0.95_)_0.95_Pb(I_0.95_Br_0.05_) and an optical bandgap of 1.58 eV, as determined from the derivative of the external quantum efficiency (EQE) shown in Figure S3 (Supporting Information). Full experimental procedures are detailed in the Supporting Information. To confirm the formation of the 2D structure following PEAI passivation, grazing‐incidence wide‐angle X‐ray scattering (GIWAXS) and grazing‐incidence diffractograms (GI‐XRD) were recorded at an incident angle of 3°, as shown in **Figure** **1**b,c. The results demonstrate that the 2D/3D perovskite films exhibit distinct 2D diffraction peaks at scattering vector q values of 3.8 nm^−1^ or angles ≈2*θ* = 4.9° which we attribute to the (002) plane of the (PEA)₂PbI₄ 2D layer, which is consistent with our recent publication^[^ ^28^ ^]^ and other reports.^[^ ^29^, ^30^, ^31^, ^32^ ^]^ Figure 1d shows the *J–V* curves of control and PEAI‐passivated PSCs in reverse scan direction. Without a passivation layer we reach a PCE of 22.0% with a *J*_SC_ of 25.0 mA cm^−2^, a *V*_OC_ of 1.12 V, and an FF of 77.1%, see Figure 1d. With an optimized PEAI passivation to form the 2D/3D heterostructure, we observe an improved PCE of 23.6% with a *V*_OC_ of 1.18 V, an FF of 81.5%, yet the *J*_SC_ dropped to 24.2 mA cm^−2^, see Figure 1d and Figure S2 (Supporting Information) for optimization details. The significant improvement in PCE comes from an improvement in *V*_OC_ and FF compared to the reference devices. To validate these findings, we provided a comprehensive statistical analysis of key parameters, including *V*_OC_, FF, and *J*_SC_, obtained from a set of 75 individual solar cell devices in Figure 1e–h, respectively. Figure 1e shows that significant *V*_OC_ improvements are observed in PEAI‐treated devices compared to the reference. Comparing the *V*_OC_ and quasi‐Fermi level splitting (QFLS) improvements in devices to improvements of PLQY of perovskite films with and without passivation layers (see Figure S4, Supporting Information), we can attribute this improvement to: i) less recombination at the perovskite/C_60_ interface, through a reduction of minority carrier concentration at the critical interface, reducing the QFLS‐*V*_OC_ mismatch as discussed previously;^[^ ^14^, ^15^, ^16^, ^17^, ^18^, ^19^, ^20^, ^21^, ^22^, ^23^, ^24^, ^25^, ^26^, ^27^, ^28^ ^]^ and ii) passivation of perovskite surface defects, which leads to a slight increase PLQY of the neat layer from 2.1% to 2.7%.

However, it is worth noting that PEAI passivation decreases the *J*_SC_ from 24.75 mA cm^−2^ (control) to 23.45 mA cm^−2^ (PEAI‐passivated) on average in agreement with previous reports_._^[^ ^20^, ^21^ ^]^ This 1.3 mA cm^−2^ drop should not be neglected, and eliminating this loss could further improve the PCE. This loss in *J*_SC_ has previously been reported as being caused by the formation of a low‐conductivity 2D passivation layer, which could lead to a shielding of the built‐in field which in turn hinders efficient charge transport.^[^ ^21^, ^22^, ^23^, ^24^, ^25^, ^26^, ^27^, ^28^, ^29^, ^30^, ^31^, ^32^, ^33^, ^34^ ^]^ However, it is notable that the FF does not decrease in proportion to the loss in *J*_SC,_ instead, it increases despite the *J*_SC_ loss, which contradicts this hypothesis. To corroborate this contradiction, we varied the PEAI concentration in the passivation treatment. We found a gradual increase of the *J*_SC_ loss with increasing PEAI concentration, yet the FF increases until a concentration of 20 mM/IPA, where the devices reach a maximum PCE. Only at even higher concentrations, above 20 mm/IPA, we observe a decrease in the FF as expected due to the limiting conductivity of the 2D phase (see Figure S2, Supporting Information). Therefore, we hypothesize that the *J*_SC_ loss at lower concentrations of PEAI (<20 mM/IPA) does not stem from a low conductivity at the interface, but is caused by a different mechanism, which we will investigate in the following.

### 2.2 Fast Hysteresis and Current Transients Measurements

**Figure 2:**
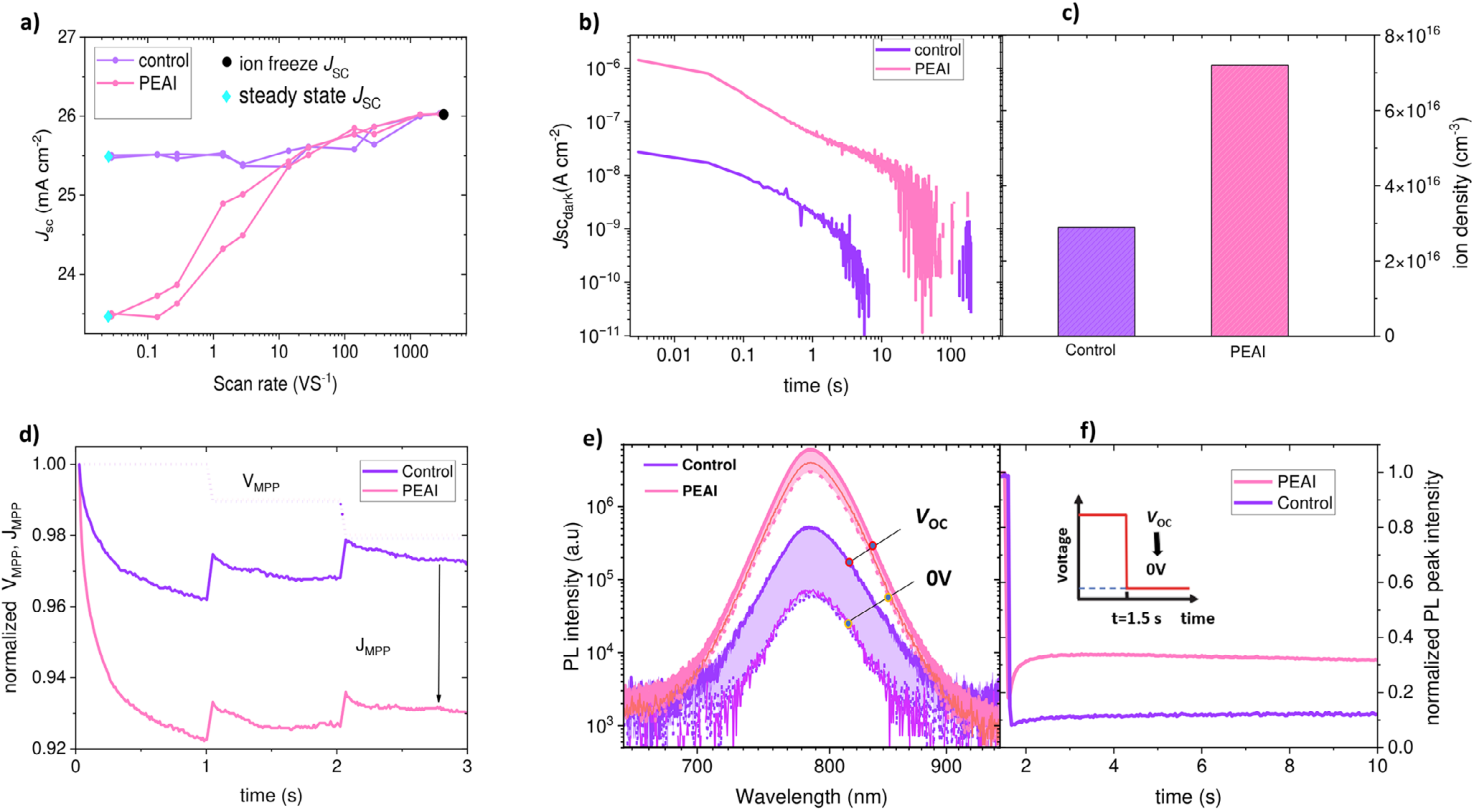
Correlating PEAI‐induced short circuit current density losses with ion density and ionic loss. a) The *J*_SC_ from fast hysteresis (FH) measurements at different scan speeds of control and PEAI‐passivated devices. b,c) Current transients from BACE measurements for both devices. d) *V*_MPP_ and *J*_MPP_ tracking of the devices showing the *J*_MPP_ decay for passivated devices on short timescales. e) The photoluminescence (PL) trace over time, taken while switching from OC to SC. f) The maximum height of the PL peaks as a function of time, where *t* = 1.5s is the moment where the bias of the device was switched from OC to SC.

To elucidate the factors contributing to the reduced *J*_SC_, we conducted and analyzed scan‐rate‐dependent *J–V* measurements, i.e., fast hysteresis (FH) assessments, on devices with and without passivation (**Figure** **2**a) as detailed in the Supporting Information. This method enables us to record *J–V* curves at very high scan speeds (up to 3000 V s^−1^), effectively surpassing the scan speed limitations of standard source measure units (SMUs). As we have previously reported,^[^ ^28^, ^29^, ^30^, ^31^, ^32^, ^33^, ^34^, ^35^, ^36^, ^37^ ^]^ this allows to analyze hysteresis due to mobile ion migration to the perovskite‐transport layer interfaces, thereby screening the built‐in field and reducing charge extraction efficiency.^[^ ^35^, ^36^, ^37^, ^38^, ^39^, ^40^, ^41^ ^]^ At high speeds, mobile ions are essentially immobilized, allowing us to assess the *J*_SC_ without the influence of ion migration, termed “ion‐freeze” *J*_SC_. Conversely, very slow scan speeds reflect the stabilized (steady state) *J*_SC_ or performance, where ions can accumulate at the interfaces, screening the built‐in field resulting in *J*_SC_ losses due to band flattening. The *J*_SC_ losses due to mobile ion redistribution are then estimated by comparing the *J*_SC_ differences between the fast and slow scans. As shown in Figure 2a, PEAI‐passivated devices experience significant current density losses of ≈2.5 mA cm^−2^ at the slowest scan speeds (0.1 V s^−1^) with respect to the fast‐scan (1000 V s^−1^), while the control device exhibited a current density loss of only ≈0.5 mA cm^−2^. This indicates that the *J*_SC_ loss in PEAI‐passivated devices is primarily driven by field screening due to ion migration, rather than by low conductivity of the 2D interlayer. These ionic *J*_SC_ losses ultimately translate to an ionic efficiency loss of ≈4.5% for PEAI‐based devices, as can be seen in Figure S5, Supporting Information.

Next, we aim to corroborate the fast‐hysteresis measurements with complementary charge extraction measurements. We estimated the density of mobile ions and their extraction time as shown in Figure 2b,c using BACE measurements in the dark following the routines reported in refs. [35, 36, 37, 38, 39, 40, 41, 42, 43] For this purpose, the cell is maintained in an “OC‐like” condition, and the displacement current is recorded when switching from *V*oc to 0 V. The displacement current, caused by mobile ions moving to transport layers or electrodes, is then integrated over time to determine the ionic density (*n*_ion_) in the active layer.

The mobile ion density is calculated using the following equation:

(1) $$n_{\mathrm{ion}}=\frac{1}{e\;d}\int_{0}^{\infty }J_{\mathrm{ion}}dt$$

where *J*_ion_ is the measured ionic current density, *e* is the elementary charge, and *d* is the device thickness. As shown in Figure 2b, the externally measured ionic displacement current is greatly enhanced in the fresh PEAI‐passivated devices compared to the control devices. Estimating *n*_ion_ reveals a fivefold increase, see Figure 2c, in the passivated device. This is consistent with the fast‐hysteresis results. Figure 2d illustrates the MPP tracking of control and PEAI‐passivated devices, highlighting a rapid initial drop in *J*_MPP_, ​ and PCE (Figure S6, Supporting Information) for PEAI‐passivated devices compared to the control. This decline is attributed to high mobile ion concentrations introduced by PEAI.^[^ ^1^, ^2^, ^3^, ^4^, ^5^, ^6^, ^7^, ^8^, ^9^, ^10^, ^11^, ^12^, ^13^, ^14^, ^15^, ^16^, ^17^, ^18^, ^19^, ^20^, ^21^, ^22^, ^23^, ^24^, ^25^, ^26^, ^27^, ^28^, ^29^, ^30^, ^31^, ^32^, ^33^, ^34^, ^35^, ^36^, ^37^ ^]^ While *V*_MPP_ remains relatively stable, the significant reduction in *J*_MPP_ ​ emphasizes the impact of mobile ions on current density. We note that side ion collection from small pixels may increase the ionic density within the active area, compared to large‐area devices.^[^ ^44^ ^]^ Nevertheless, using identical active areas, we can quantify the impact of PEAI on the ionic density in control and passivated specimens.

To figure out if we indeed suffer from charge extraction losses, we measured the PL of complete devices from the cell as a function of time after switching the voltage from *V*oc to 0 V while the devices remained under 1 sun equivalent continuous illumination.^[^ ^34^, ^35^, ^36^, ^37^, ^38^, ^39^, ^40^, ^41^, ^42^, ^43^, ^44^, ^45^, ^46^ ^]^ As shown in Figure 2d, when the device bias switched from *V*oc (solid lines) to 0 V (dotted lines) at around *t* = 1.5 s, the PEAI‐passivated device exhibited less PL quenching compared to the control. Additionally, after the initial quench, the PL peak quickly rises again (Figure 2e) despite the device remaining under short‐circuit conditions. We note that although, the full “quench of the PL” after the switch cannot be fully resolved due to a limited time resolution (≈100 ms), this effect is more significant for the PEAI device compared to the control sample. This indicates a time‐dependent ion migration within the device, leading to charge accumulation, which impedes the efficient extraction of charges from the active layer to the transport layer. These findings reinforce the conclusion that ionic movement is a key contributor to the observed current losses. Nevertheless, with the PEAI passivation PLQY is significantly higher than on non‐passivated samples, which indicates that the PEAI treatment has indeed the desired passivating effect, in agreement with the large increase of *V*oc.^[^ ^10^, ^11^, ^12^, ^13^, ^14^, ^15^, ^16^, ^17^, ^18^, ^19^, ^20^, ^21^, ^22^, ^23^, ^24^, ^25^, ^26^, ^27^, ^28^, ^29^, ^30^, ^31^, ^32^, ^33^, ^34^, ^35^, ^36^, ^37^, ^38^, ^39^, ^40^, ^41^, ^42^, ^43^, ^44^, ^45^, ^46^, ^47^, ^48^, ^49^ ^]^

To generalize our findings, we studied the *J*_SC_ loss of control and PEAI‐passivated devices in another high‐performance perovskite composition, namely triple cation perovskite (Cs_0.05_ (FA_0.98_MA_0.02_)_0.95_Pb(I_0.98_Br_0.02_)_3_) (“98:02 TC”). As shown in Figure S7 (Supporting Information), we observe the same picture, suggesting that the formation of the 2D layer and/or the PEAI‐salt itself increases the density of mobile ions in the device, independently of the composition of the 3D perovskite layer. To this end, we believe that this is predominantly due to FA^+^/MA^+^/Cs^+^ and I^−^ ions, which would be released during 2D perovskite formation from the 3D perovskite following:

(2) $$\mathrm{APb}I_{3}+2\times \mathrm{PEAI}\to \mathrm{PE}A_{2}\mathrm{Pb}I_{4}+A^{+}+I^{-}$$

with A being FA, MA or Cs. Direct deposition of the 2D layer from orthogonal solvents could minimize this.^[^ ^50^ ^]^ However, recent publications also indicate that the PEA^+^ can migrate at elevated temperatures and under illumination, causing ionic losses which increase upon operation.^[^ ^27^ ^]^

## 3 Mitigation Strategies of PEAI‐Induced *J*_SC_ Loss

In the following, we propose and demonstrate strategies to control PEAI‐induced ionic *J*_SC_ losses by incorporating ultrathin layers of ABS or EDAI_2_ salts between the PEAI and the perovskite, as illustrated schematically in Figure S8a (Supporting Information).

EDAI₂ hereby can form strong H‐bonds both with PEAI and as well as the perovskite surface due to its amino groups at both ends. This can immobilize PEA^+^, I^−^ or even FA^+^.^[^ ^32^, ^33^, ^34^, ^35^, ^36^, ^37^, ^38^, ^39^, ^40^, ^41^, ^42^, ^43^, ^44^, ^45^, ^46^, ^47^, ^48^, ^49^, ^50^, ^51^, ^52^ ^]^ Similarly, the SO₃⁻ functional groups of ABS, being electron‐rich, can form strong coordination bonds with the NH₃⁺ groups of PEA^+^ and the perovskite surface.^[^ ^53^ ^]^ Again, this interaction could create an effective anion migration barrier at the perovskite interface, suppressing ionic species migration into the perovskite layer. Following the introduction of ABS and/or EDAI₂, we conducted grazing‐incidence wide‐angle X‐ray scattering (GIWAXS) (Figure S8b–e, Supporting Information), and grazing‐incidence XRD (GIXRD) measurements (Figure S9, Supporting Information). In all cases, we observe the formation of a 2D phase. In the case of EDAI_2_ and ABS‐based devices, the 2D formation was even further confined to the surface, highlighting the suppressed migration, as evident from incident angle‐dependent GIXRD, see Figure S8f (Supporting Information), albeit the observed difference to the bare PEAI is small.

**Figure 3:**
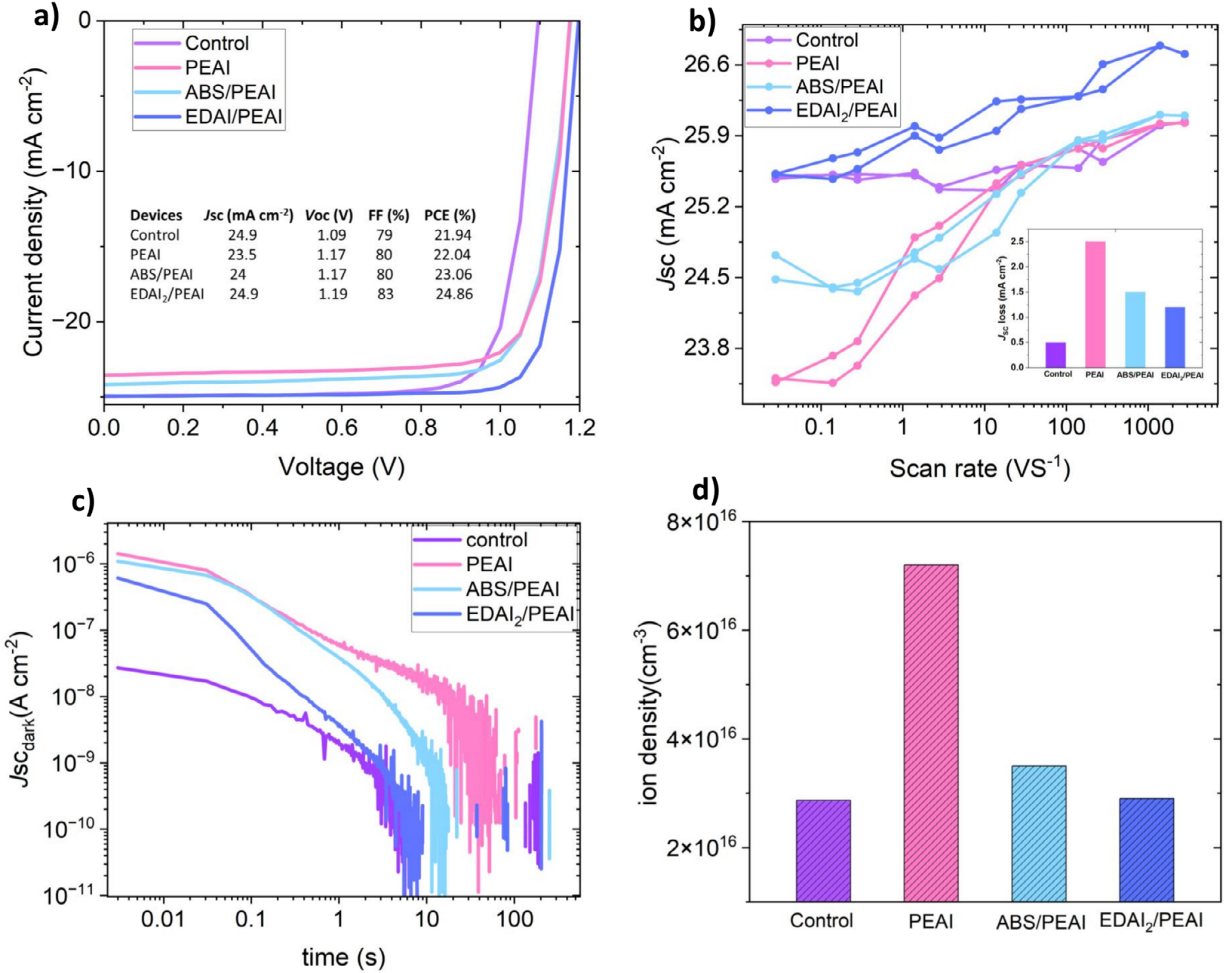
Mitigating PEAI‐induced short circuit current density losses with ABS and EDAI_2_ interlayers. a) *J*–*V* characteristics of the PSCs of champion devices. b) Absolute PL spectra and the corresponding PLQY. b) The *J*_SC_ from fast hysteresis (FH) measurements at different scan speeds. The inset shows the *J*_SC_ loss obtained from FH measurements for all devices_._ All the remaining FH data can be found in Figure S13 (Supporting Information). c,d) Current transients from BACE measurements and corresponding integrated current which is assigned to the mobile ion density.

Incorporating ABS or EDAI_2_ salts improves the *J*_SC_ significantly compared to devices treated solely with PEAI, see **Figure** **3**a. Especially the EDAI_2_/PEAI bilayer passivation enables *J*_SC_ values en‐par with un‐passivated devices, while at the same time improving *V*oc and FF significantly. This translated in a champion efficiency of 24.86% for the EDAI_2_/PEAI bilayer passivation. The statistical distribution shown in Figure S10 (Supporting Information) further confirms these findings, highlighting a significant improvement of *V*oc and FF, which together contribute to the enhanced PCE. The *J*_SC_, remains comparable between the control and EDAI₂/PEAI passivated devices, indicating that the bilayer passivation with EDAI_2_ effectively reduced PEAI‐induced *J*_SC_ drop. To clearly show that reduced ionic‐losses enable this improvement we conducted fast hysteresis measurements. Figure 3b shows the short‐circuit current density as a function of the scan speed of control and PEAI, ABS/PEAI, and EDAI_2_/PEAI treated devices. Estimated *J*_SC_ losses due to mobile ions are suppressed from ≈2.5 mA cm^−2^ (PEAI‐passivated) to ≈1.4 mA cm^−2^ and ≈1.1 mA cm^−2^ for ABS/PEAI and EDAI_2_/PEAI treated devices, respectively (see inset in Figure 3b). This corresponds to about a 40% and 55% reduction in *J*_SC_ losses when incorporating a layer of ABS and/or EDAI_2_ salts between the PEAI and perovskite films, respectively, compared to the PEAI‐passivated device. To further corroborate the fast‐hysteresis measurements, we also performed BACE measurements (Figure 3c,d) on the same devices. The result reveals a reduced mobile ion density for the ABS/PEAI and EDAI_2_/PEAI passivation compared to the PEAI passivated devices. Similarly, to before, we double‐checked this on a second perovskite composition, again observing the same result that ABS/PEAI and EDAI_2_/PEAI systems reduce the mobile ion density and *J*_SC_ losses compared to PEAI‐based passivation (see Figure S11, Supporting Information). We measured the work function using Kelvin probe spectroscopy (Figure S12, Supporting Information) but find only minimal variation after bilayer passivation compared to PEAI only, suggesting that energetic alignment plays a minimal role, while ionic losses dominate observed trends.

## 4 Device Stability

**Figure 4:**
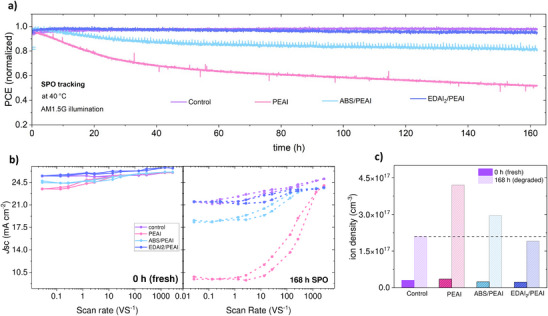
a) SPO tracking under 1 sun equivalent illumination in ambient atmosphere, at a temperature of 40 °C of the encapsulated control versus the devices treated with PEAI, ABS/PEAI, and EDAI_2_/PEAI. b) *J*_SC_ obtained from fast hysteresis (FH) measurements of the devices before and after SPO tracking. c) integrated on density of the devices before and after SPO tracking obtained from BACE measurements. All fast hysteresis data of the fresh and SPO‐tracked PEAI devices can be found in Figure S18 (Supporting Information). The measurements demonstrate that the observed degradation in the PEAI passivated device stems largely from ionic losses while the ion‐freeze *J*_SC_ from fast hysteresis after SPO tracking remains nearly unchanged.

To compare the long‐term stability devices without and with PEAI, ABS/PEAI, and EDAI_2_/PEAI treatment, we encapsulated devices and monitored their performance in an ambient atmosphere under constant 100 mW cm^−2^ illumination at a temperature of 40 °C. As shown in **Figure** **4**a, devices passivated with PEAI suffered from significant degradation in PCE to about 50% of its initial PCE after only 160 h. This accelerated degradation stems, as we will show later from the high density of mobile ions. Treating the PEAI/perovskite interface with EDAI_2_ salts, i.e., utilizing the EDAI_2_/PEAI bilayer, improves the remaining efficiency to 95% after 160 h. This corresponds to about 42% long‐term stability enhancement compared to PEAI‐only devices. ABS/PEAI‐treated devices, also show an increased stability with respect to the PEAI‐passivated device, albeit smaller than for EDAI_2_/PEAI bilayers as expected from the initial ion density.

To assess whether the stability correlates with the initial mobile ion density, we performed fast hysteresis and BACE measurements on fresh (before SPO tracking) and after 160 h SPO tracking. Recently, our group extensively studied the role of mobile ions in the light‐induced performance degradation of PSCs under various external stress conditions, demonstrating that mobile‐ion‐induced field screening is the primary reason for illumination‐induced PCE degradation.^[^ ^36^ ^]^ Similarly, the correlation between peak hysteresis and device stability across different perovskite systems has been explored by other researchers, providing further insights into the factors affecting long‐term performance.^[^ ^54^, ^55^ ^]^ The FH measurements for PEAI passivated device taken initially and after the SPO tracking (Figure 4b) reveal a nearly unchanged ion‐freeze *J*_SC_, but significantly increases *J*_SC_ loss from ≈2.5 mA cm^−2^ (fresh) to ≈14 mA cm^−2^ (160 h SPO tracking), dominating the observed performance degradation. For ABS/PEAI‐treated and EDAI_2_/PEAI‐treated devices, this ion‐induced *J*_SC_ loss is effectively reduced after 160 h. Evaluating the ionic density from BACE measurements corroborates this picture: PEAI‐passivated devices exhibit the highest ion density, after 160 h, while ABS/PEAI and EDAI_2_/PEAI‐passivated devices exhibit reduced ion density even after 160 h (see Figure 4c) in agreement with the fast‐hysteresis measurements. Note, that the ionic density of EDAI_2_/PEAI treated devices after 160 h of aging is even below non‐passivated devices. Moreover, the bilayer‐passivated devices (EDAI₂/PEAI and ABS/PEAI) maintain excellent stability at 60 °C (see Figure S15, Supporting Information), while PEAI‐only passivated devices degrade significantly faster, confirming that mobile ion migration is the primary cause of Jsc losses and instability. This accelerated degradation at elevated temperatures further supports our findings and highlights the superior thermal stability of our bilayer passivation strategy.

**Figure 5:**
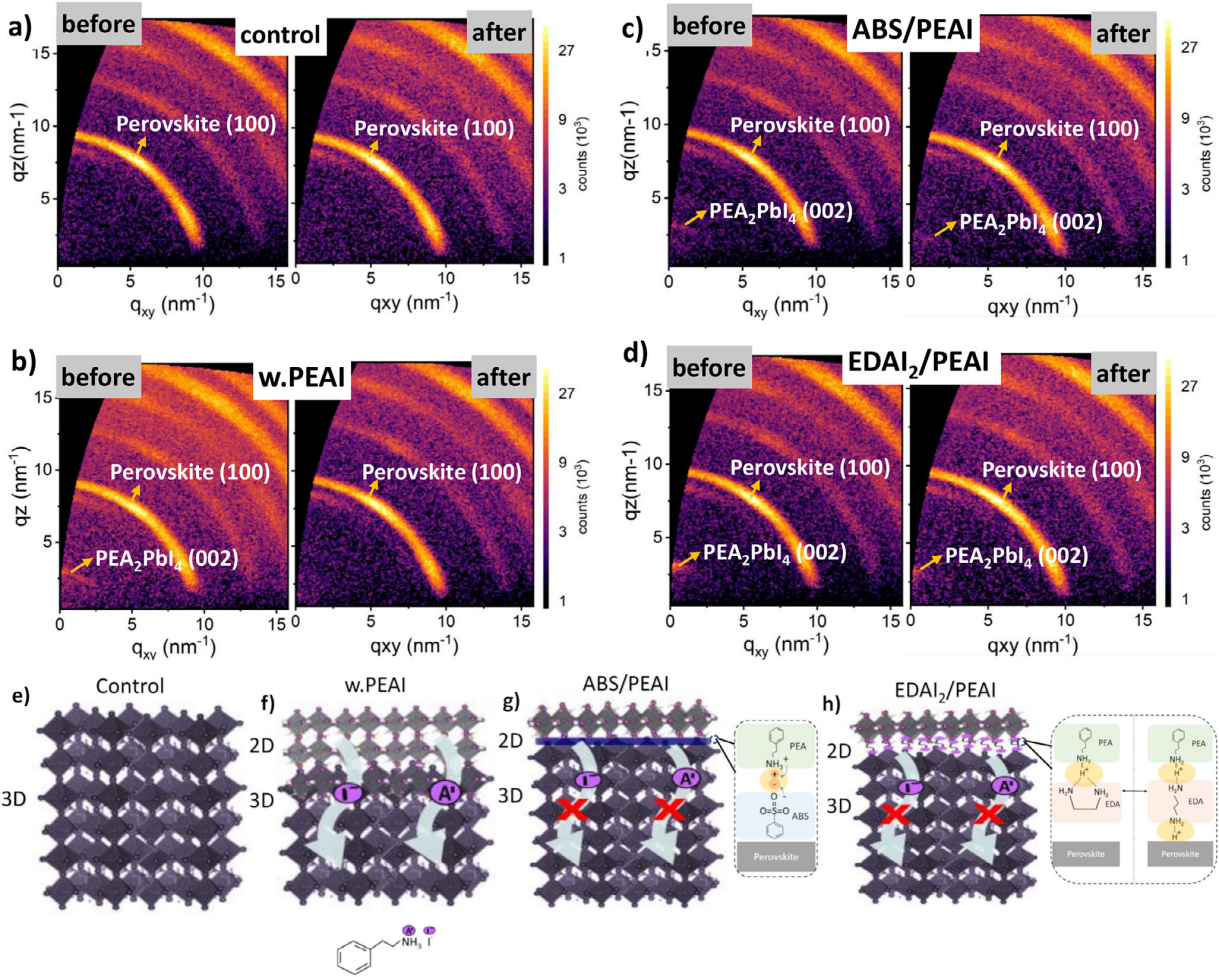
a−d) 2D GIWAXS images for control, PEAI, ABS/PEAI, and EDAI2/PEAI‐based 2D/3D perovskite films. The images depict fresh films and those subjected to 168 h of 1 sun illumination light‐induced aging. e−h) Schematics summarizing PEAI‐2D anion and cation migration pathways in 2D/3D perovskite stacks. In the case of PEAI, upon spin‐coating of the PEAI dissolved in IPA onto the 3D perovskite, both PEA^+^ cations and I^−^ appears to penetrate the 3D perovskites and induce additional ions into the bulk, possibly explaining the absence of PEA_2_PbI_4_ diffractions in the GIWAXS images after prolonged aging.

To investigate the structural integrity of the 2D perovskite interlayers after degradation, we recorded grazing incidence wide‐angle X‐ray scattering (GIWAXS) images of fresh and 168 h aged passivated and nonpassivated films (**Figure** **5**a–d). In PEAI‐passivated films we observe an almost complete disappearance of the PEA_2_PbI_4_ (002) related diffraction peaks, suggesting poor structural stability of the 2D/3D heterojunction (Figure 5b). On the other hand, ABS/PEAI and EDAI_2_/PEAI passivation enable a stable 2D/3D heterojunction even after 168 h of aging (Figure 5c,d).

Overall, we observe a poor structural instability of the bare 2D/3D interface using only PEAI, resulting in i) high ionic density in fresh devices, ii) accelerated degradation, iii) disappearance of the 2D phase upon prolonged aging, iv) increased ionic losses with increased ionic density after aging. The root cause seems to be the high mobility of PEA^+^ into the 3D perovskite bulk (see Figure 5f). Indeed, Moral et al. recently found a high mobility of PEA^+^ cations indicating that even relatively large cations can migrate through the 3D perovskite.^[^ ^56^ ^]^ Furthermore, Perini et al. investigated the thermal stability of PEAI bulky cation layers and discovered that thermal stressing of PEAI passivated interface leads to the diffusion of bulky cation cations into the perovskite layer, altering the structure and crystallinity of the interface.^[^ ^26^ ^]^ This diffusion consequently led to a significant drop in performance within the first 200 h of maximum power point tracking (MPPT), even at temperatures of 55 °C. Moreover, Park et al. reported that PEA‐based 2D perovskite films exhibited thermal instability after being aged at 85 °C for 2 h. Again, they attributed this instability to the diffusion of PEA cations into the bulk perovskite.^[^ ^27^ ^]^ On the contrary, we do not observe structural instabilities or increased ionic densities for ABS/PEAI, and EDAI_2_/PEAI ‐treated devices. This suggests that the R‐SO₃⁻ functional groups in ABS, with their high electron density, exhibit a strong tendency to form coordination bonds,^[^ ^53^ ^]^ establishing robust interactions with the NH₃⁺ groups of PEAI and the perovskite surface (Figure 5g). This interaction effectively anchors the passivation layer to the perovskite surface. Moreover, the strong coordination between SO₃⁻ and NH₃⁺ groups create a physical and electrostatic barrier at the perovskite surface, which hinders the migration of anions, such as iodide (I⁻), and NH₃⁺ into the perovskite layer. EDAI_2_/PEAI‐passivated devices showed even higher structural stability of the 2D/3D interface. This is corroborated by a recent study by Huang et al., who proposes that ethylene diamine (EDA) can act as an interfacial ligand confinement agent enabling well‐controlled 2D/3D perovskite interfaces.^[^ ^32^ ^]^ This is due to the two amino groups of EDA, which can bridge the 2D and 3D perovskites, and minimize the potential cation exchange between the 2D and 3D perovskite.^[^ ^57^ ^]^ In other words, this interaction can effectively block the ion migration channels present in PEAI‐only treated films (see illustration Figure 5h). This then enables to lower the mobile ion density in fresh devices minimizing ionic losses and increasing performance, while at the same time enabling highest device stability.

Incorporating bulky cations that do not readily form 2D perovskites or exhibit limited migration within the perovskite lattice presents another viable strategy to minimize ionic losses and mitigate instabilities commonly associated with 2D passivation approaches.^[^ ^58^ ^]^ However, a comprehensive exploration of these effects falls beyond the scope of this work.

## 5 Conclusion

In conclusion, our findings reveal that while PEAI effectively passivates surface defects and enhances open‐circuit voltage (*V*_OC_) and fill factor (FF), it also leads to significant *J*_SC_ losses. Fast‐hysteresis measurements prove that the *J*_SC_ loss is due to comparatively high mobile ion concentration, rather than the low conductivity of the 2D phase. Correlating these data with the results from various transient electronic measurements we identify a high ion density induced by the PEAI, causing ≈2.5 mA cm^−2^ ionic *J*sc losses already in fresh device. We then show that PEAI further leads to an accelerated degradation under operational conditions compared to control devices. The primary reason is an even further increase of ion density leading to an ionic *J*sc loss of (≈14 mA cm⁻^2^) after 160 hours, while ion‐freeze currents are unaffected. This is further corroborated by GIWAX diffractograms, which reveal a poor structural stability of the 2D/3D heterostructure. Aiming to reduce PEAI‐induced mobile ions and the corresponding *J*_SC_ loss_,_ we then introduced ABS and EDAI_2_ interlayers between PEAI and perovskite interfaces. This led to a reduction of the PEAI‐induced mobile ion density and minimized ionic *J*sc losses by ≈40% and ≈55% respectively for EDAI_2_ and ABS. Overall, these interlayers effectively stabilized the 2D/3D heterostructure and inhibited the formation of a high mobile ion density. As a result, the bilayer passivation strategy not only mitigated the *J*_SC_ losses but also enhanced the overall device stability under operational conditions paving the way for more stable and efficient perovskite solar cells.

## Conflict of Interest

The authors declare no conflict of interest.

## Supporting information



Supporting Information

## Data Availability

The data that support the findings of this study are available from the corresponding author upon reasonable request.
