# Supplementary material for: Mitigating Mobile‐Ion‐Induced Instabilities and Performance Losses in 2D Passivated Perovskite Solar Cells
Source: Adv Mater. 2025 May 9;37(30):2501588. doi: 10.1002/adma.202501588 (PMC12306393; doi:10.1002/adma.202501588)
Supplement: Supplementary file 1 — Supporting Information [file ADMA-37-2501588-s001.pdf]

ADVANCED MATERIALS

Supporting Information

for *Adv. Mater.*, DOI 10.1002/adma.202501588

Mitigating Mobile-Ion-Induced Instabilities and Performance Losses in 2D Passivated Perovskite Solar Cells

Biruk Alebachew Seid, Sercan Ozen, Andrés-Felipe Castro-Méndez, Dieter Neher, Martin Stolterfoht and Felix Lang**

Supporting Information (SI)

Mitigating mobile-ion-induced instabilities and performance losses in 2D passivated perovskite solar cells

Biruk Alebachew Seid*[1], Sercan Ozen[1], Andrés-Felipe Castro-Méndez[1], Dieter Neher[1], Martin Stolterfoht[2], and Felix Lang*[1]

[1] Physik und Optoelektronik weicher Materie, Institut für Physik und Astronomie, Universität Potsdam, Germany

[2]Electronic Engineering Department, The Chinese University of Hong Kong, Hong Kong SAR, Chinaotsdam, China

*Corresponding authors: E-mail: felix.lang.1@uni-potsdam.de, seid@uni-potsdam.de

Experimental Methods

Preparation of $Cs_{0.05}(MA_{0.05}FA_{0.95})_{0.95}Pb(I_{0.95}Br_{0.05})$ triple-cation perovskite solutions

The perovskite precursor solution was prepared following the protocol outlined by Seid et al.[1] Specifically, PbI_2 (1797.94 mg), $PbBr_2$ (578.06 mg), MABr (79.96 mg), and FAI (567.98 mg) were dissolved in a DMF/DMSO solvent mixture (4:1 v/v) and stirred to yield a 1.5 M solution of $FAPbI_3$ and $MAPbBr_3$. Subsequently, a CsI (1.5 M) solution along with MACl (20 mol%) in DMSO was added to the mixture to complete the formulation.

Preparation of $Cs_{0.05}(MA_{0.02}FA_{0.98})_{0.95}Pb(I_{0.98}Br_{0.02})_3$ triple-cation perovskite solutions

The solution was prepared by adopting the procedure reported by Zhen Li et al. [2] a PbI_2 (909.00 mg), FAI (276.06 mg), MABr (3.68 mg), CSI (22.47 mg), and MACl (18.11 mg) were mixed in a DMF/DMSO solvent mixture (5/1 v/v) and stirred for 4 h at 60 °C to form a 1.73 M ink of $Cs_{0.05}(MA_{0.02}FA_{0.98})_{0.95}Pb(I_{0.98}Br_{0.02})_3$ perovskite solutions.

Preparation of 2D perovskite solutions

The passivation layers were prepared using high-purity materials from Sigma-Aldrich: PEAI (98%), $EDAI_2$ (>98%), and ABS (>99.9%). For PEAI passivation, 3.5 mg of PEAI was dissolved in 1 mL of isopropanol (IPA), sonicated for 30 minutes, and spin-coated

onto cooled perovskite substrates at 5000 rpm for 40 seconds without additional annealing. The $EDAI_2$ passivation layer was prepared by dissolving 2 mg of $EDAI_2$ in a 1:1 (v/v) mixture of IPA and toluene, followed by 30 minutes of ultrasonic treatment, spin-coating at 5000 rpm for 40 seconds, and annealing at 100°C for 10 minutes. For ABS passivation, 1 mg of ABS was dissolved in 1 mL of ethanol, sonicated for 30 minutes, spin-coated at 5000 rpm for 40 seconds, and annealed at 100°C for 35 seconds. For the bi-layered passivation, a subsequent PEAI layer was deposited onto the cooled $EDAI_2$- or ABS-passivated perovskite films using the same 5000 rpm, 40-second spin-coating process without additional annealing.

Device Fabrication

Planar inverted perovskite solar cells were fabricated with the following layer sequence: glass/ITO/MeO-2PACz/$Cs_{0.05}(MA_{0.05}FA_{0.95})_{0.95}Pb(I_{0.95}Br_{0.05})_3$/$C_{60}$/BCP/Cu. Initially, patterned ITO-coated glass substrates underwent sequential cleaning in an ultrasonic bath using acetone, Hellmanex (3% in deionized water), deionized water, ethanol, acetone, and isopropanol for 15 minutes each. Following this, the substrates were exposed to ultraviolet ozone for 30 minutes to enhance surface activation before being transferred into a nitrogen-filled glovebox.

The MeO-2PACz layer was spin-coated from a 1 mmol mL^{-1} ethanol solution at 3000 rpm for 30 seconds and annealed at 100 °C for 10 minutes. After cooling to room temperature, the triple-cation perovskite solution was spin-coated at 4000 rpm for 40 seconds, with an acceleration time of 5 seconds. 7 s prior to the end of the spin-coating process, 300 μL of chlorobenzene was applied as an antisolvent, followed by annealing the perovskite film at 100 °C for 1 hour. A similar method was employed for the 98:02 triple-cation PSCs, except the film was annealed at 110 °C for 20 minutes, and 250 μL of chlorobenzene was dispensed 12 seconds before the spin-coating ended.

The passivation layers were prepared by spin coating the dissolved solution containing 2Ds onto the cooled perovskite substrates at 5000 rpm for 40 seconds. Following this step, the samples were placed in an evaporation chamber where 30 nm of C_{60} (deposited at 0.3 Å/s), 8 nm of BCP (deposited at 0.3 Å/s), and 100 nm of copper (Sigma-Aldrich, deposited at 0.6 Å/s) were sequentially evaporated under a high vacuum (10^{-7} mbar).

Device Characterizations

Current density-voltage characteristics: J–V characterization of Solar cells was measured at room temperature in an N_2 glove box with a source meter (Keithley 2400) using a solar simulator (Oriel class AAA Xenon lamp) at a 100 mW cm^{-2} illumination (AM 1.5 G). The light intensity was monitored simultaneously with a Si photodiode. The temperature of the cell was fixed to 25 °C and a voltage ramp of 20 mV/s was used. The device area was 0.06 cm^2 and the active area was defined by a metal mask aperture.

External quantum efficiencies (EQE): External quantum efficiencies were measured as a function of wavelength from 300 nm to 1100 nm with a step of 5 nm using a custom-built small spot EQE system. The beam size was 0.5 mm^2, which is smaller than the active area (~0.06cm^2)[3].

FH measurements: Fast *J–V* curves were obtained by applying a triangular voltage pulse to the solar cells, beginning near the *V*oc, followed by a reverse sweep from *V*oc to −0.1 V and a forward sweep from −0.1 V back to *V*oc, at varying frequencies or scan speeds (V s−1), using equipment developed by FastChar UG[4]. The holding time at *V*oc was set to be five times longer than the total duration of the voltage sweep. The voltage response of the cell was captured using an oscilloscope, with an external load resistance of ≤10 Ω, and the voltage pulse was generated using a function generator paired with a custom-built power amplifier (4×). Although the hardware and measurement protocols differed, the testing conditions matched those used for standard *J–V* measurements, as described earlier. To validate the fast-hysteresis (FH) results at lower scan speeds (10–100 mV s^{-1}), standard *J–V* measurements were conducted on the same cells, yielding performance metrics nearly identical to those obtained with the FH setup.

BACE Measurements: In dark BACE, the device was initially held at a voltage close to the open-circuit voltage, where the injected charge equals the short-circuit current. After a pre-set delay time, a bias of 0 V was applied to extract the injected and capacitive charge in the device. The delay times for the fresh devices were chosen to be typically five times longer than the extraction time of charges observed under the collection bias (typically ~5–10 s) to allow ionic charges to distribute throughout the active layer. After ~30 s, the voltage was switched to 0 V to extract the charge carriers. The current transients were measured with a Keithley 2400 using

a home-built LabView program. Finally, the extracted charge was obtained by integrating the current transient and the charge carrier density by dividing the total charge by the elementary charge and the cell volume. This process was repeated on the devices, immediately after MPPT.

Absolute Photoluminescence Measurements: Excitation for the PL measurements was performed with a 520 nm CW laser (Insaneware) through an optical fiber into an integrating sphere. The intensity of the laser was adjusted to a 1 sun equivalent intensity by illuminating a 1 cm^2 size perovskite solar cell under open circuit conditions. A second optical fiber was used from the output of the integrating sphere to an Andor SR393iB spectrometer equipped with a silicon CCD camera (DU420A-BR-DD, iDus). The system was calibrated by using a calibrated halogen lamp with specified spectral irradiance, which was shone into to integrating sphere. A spectral correction factor was established to match the spectral output of the detector to the calibrated spectral irradiance of the lamp. The spectral photon density was obtained from the corrected detector signal (spectral irradiance) by division through the photon energy (hf), and the photon numbers of the excitation and emission were obtained from numerical integration using Matlab. [5]

Voltage-dependent Photoluminescence measurements: Voltage-dependent photoluminescence (PL) measurements were carried out by illuminating a defined pixel area of the perovskite solar cell with a 520 nm continuous wave (CW) laser. During illumination, an external voltage bias (e.g., 0 V or the open-circuit voltage, Voc) was applied to the cell using a Keithley 2400 SourceMeter. To ensure that the detected emission was solely from the intended active pixel area, the cells were carefully masked to exclude photoluminescence contributions from non-active regions. The time-resolved PL spectra were collected using an Andor Solis system equipped with a high-sensitivity silicon (Si) detector, allowing for precise spectral acquisition throughout the measurement. Each experimental condition was repeated multiple times to verify the reproducibility and consistency of the obtained results. The repeated measurements confirmed the stability and reliability of the voltage-dependent PL response of the active pixel under varying bias conditions.

MPP tracking: MPP tracking was performed in an ambient atmosphere by placing encapsulated devices under a 1-sun equivalent white LED irradiation for several days. With a Botest multichannel analyzer system (Botest Systems GmbH, EMU-8/ v2.3) with a constant applied voltage (initial VMPP) using white light LED (3000K Cree CXB3590) illumination providing a 1 sun equivalent intensity by matching the initial current of the cell to the J_{SC} under

AM1.5G illumination. The temperature during the tracking was T = 40 °C, and the measurements were performed in an ambient atmosphere on encapsulated cells.

GIWAXS: Grazing incidence wide angle X-ray scattering (GIWAXS) data were collected by using Empyrean, Malvern Pananalytical. Copper K-α X-rays directed onto the sample via collimating mirror and scattered X-rays were collected by PiXel3D detector (256 * 256 pixels of 55 µm) at a distance of 31.6 mm to the sample position. Calibration of sample-to-detector distance and detector tilts was performed using Cr_2O_3 as a calibrant, processes through the PyFAI library. Lastly the measured GIWAXS data were converted to q-space by performing azimuthal angle integration using PyGIX, facilitating a direct analysis of the structural features in reciprocal space.

Supplementary Figures

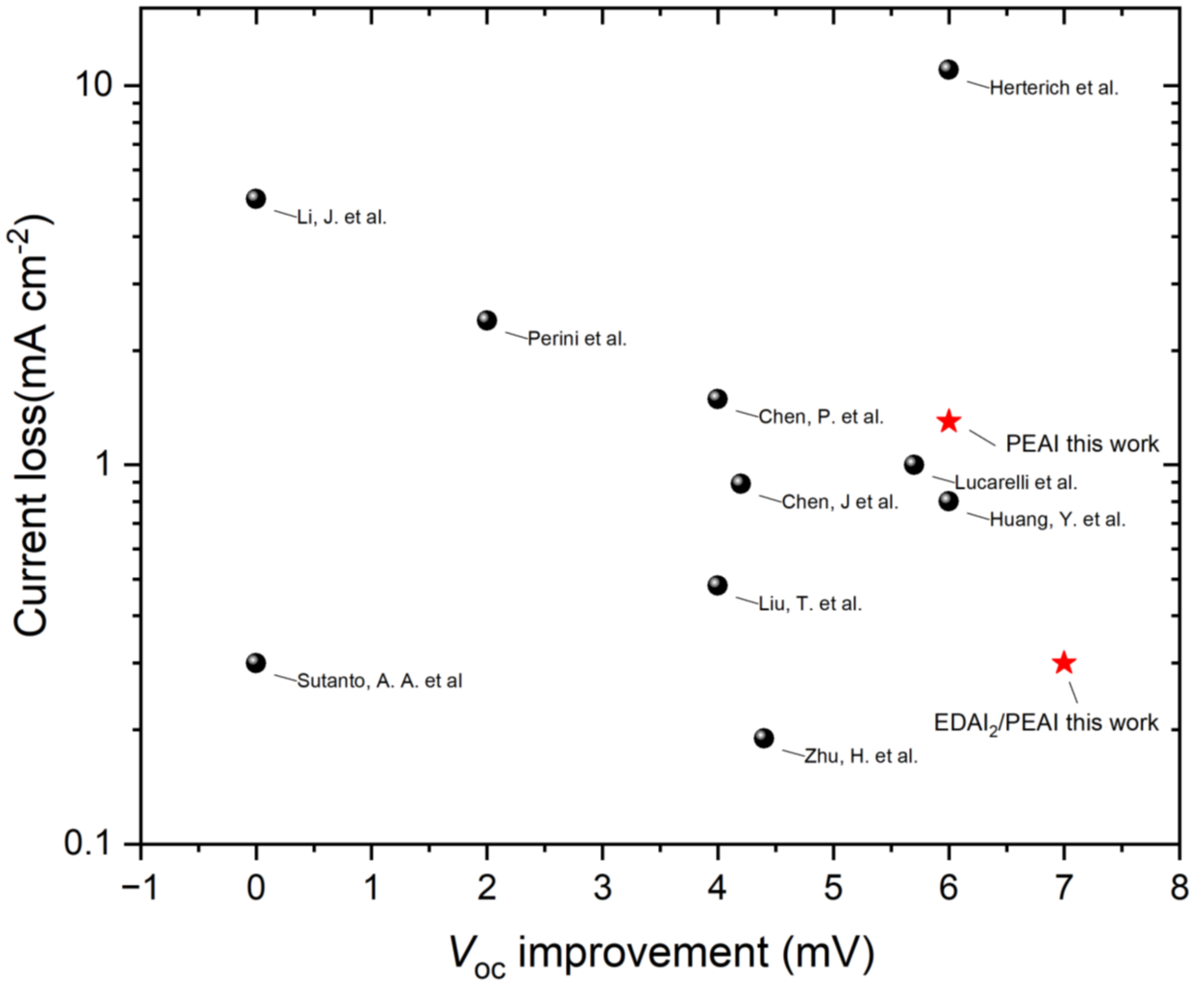

Figure S1. Comparative analysis of the current loss versus *V*oc improvement for various studies incorporating phenylethylammonium iodide (PEAI) as a passivating agent. The data points represent results from previous works[6–15], highlighting a trade-off between J_{SC} loss and *V*oc enhancement. The red stars correspond to the results of our work, demonstrating the improvements achieved in this study.

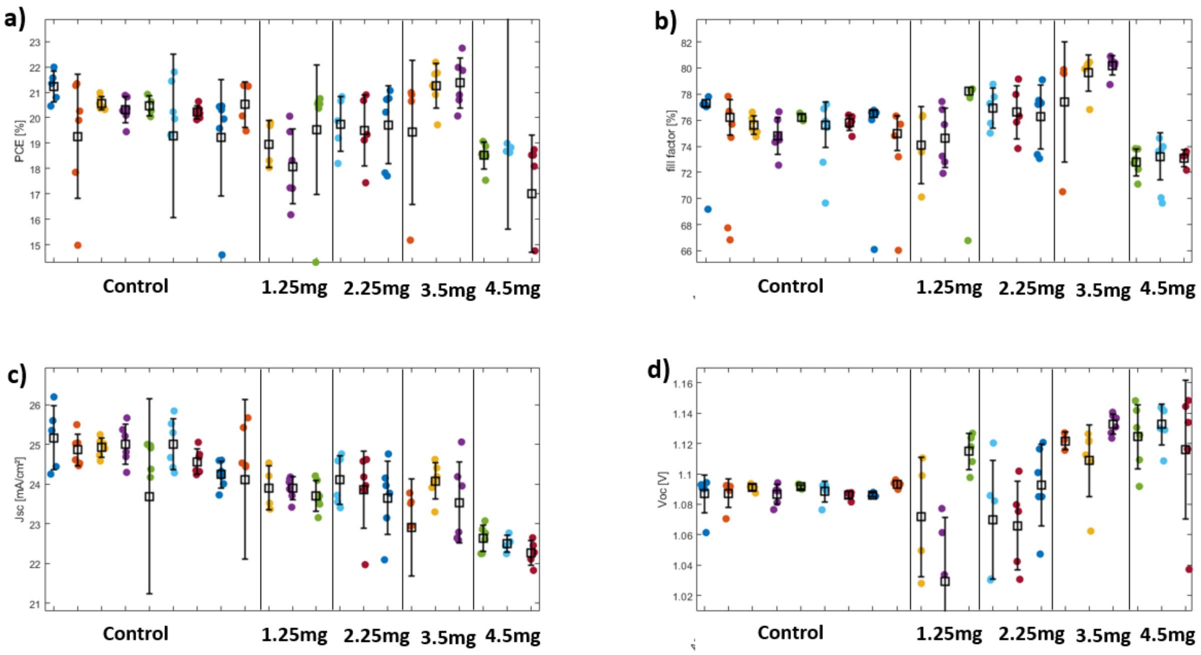

Figure S2. The distribution of key photovoltaic (PV) parameters, including open-circuit voltage (VOC), short-circuit current density (J_{SC}), fill factor (FF), and power conversion efficiency (PCE) for control and PEAI-passivated solar cells with different concentrations. These measurements were conducted across six pixels for each of the 21 fabricated devices.

The results indicated that while the passivation with PEAI improved the FF, an undesirable reduction in J_{SC} was observed as the concentration of PEAI increased. This loss in J_{SC} occurred across all tested concentrations of PEAI, suggesting that despite the enhancement in the FF, the higher PEAI concentrations negatively affected the charge collection or transport within the device.

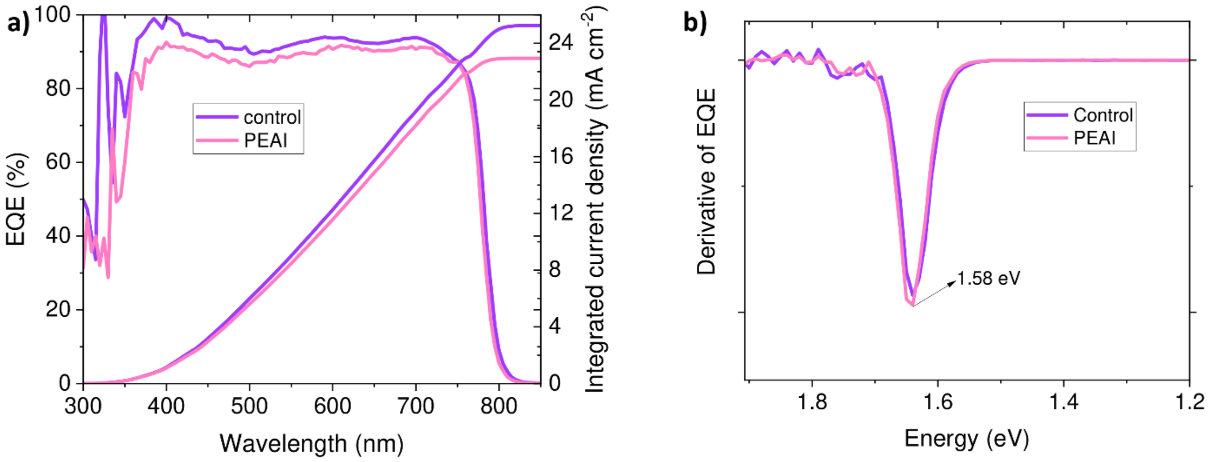

Figure S3: a) A direct comparison of external quantum efficiency (EQE) measurements of PEAI-passivated and non-passivated cells, which shows a lower integrated current density (J_{SC}) in PEAI devices. b) shows d(EQE)/dλ versus wavelength confirming a bandgap of 1.58 eV.

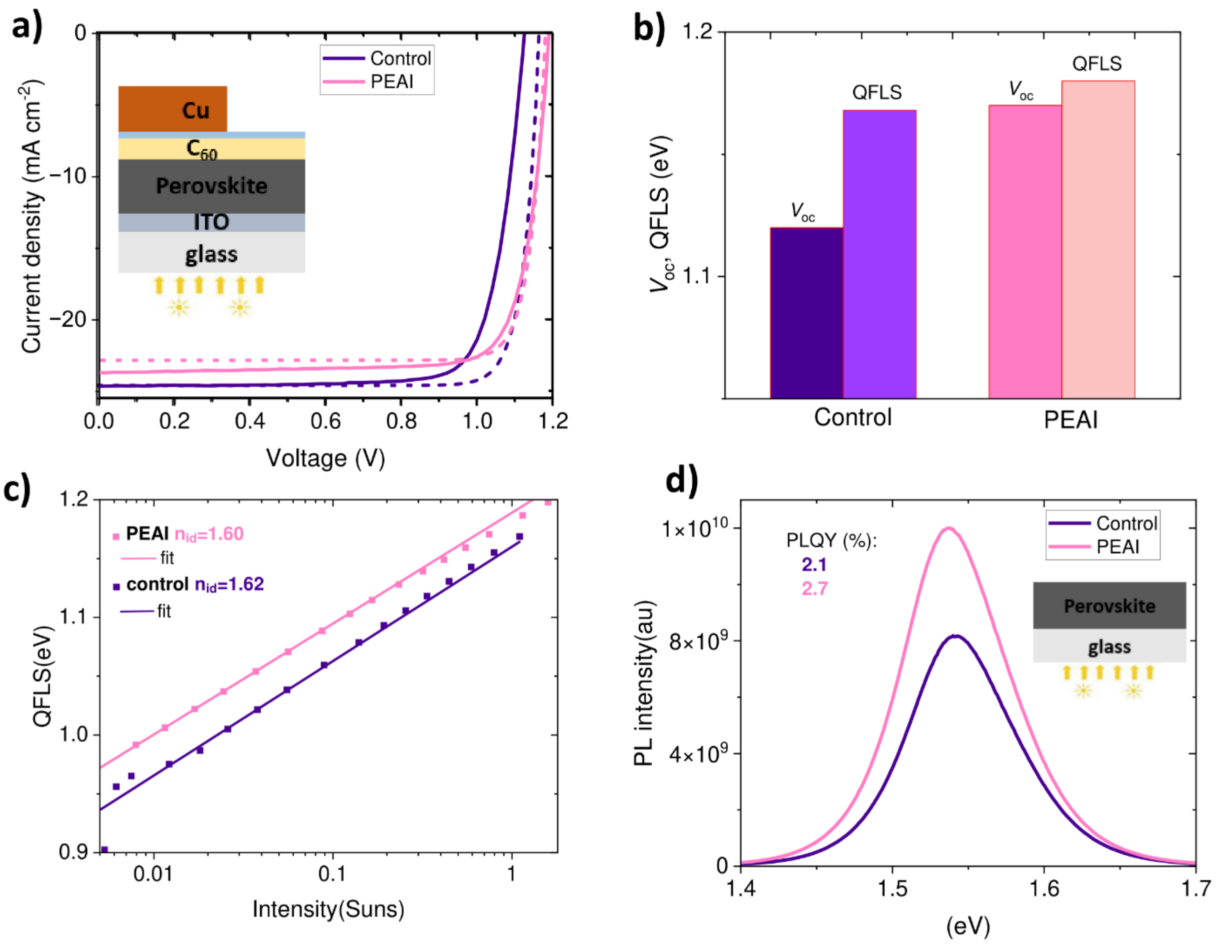

Figure S4: a, b) *JV* and pseudo-*JV* characteristics obtained from PL measurements of perovskite solar cell devices with and without PEAI, which form a 2D perovskite layer on the surface. c) Intensity-dependent quasi-Fermi level splitting and the corresponding ideality factors. d) Photoluminescence at a 520 nm excitation wavelength for both control and PEAI-passivated films on glass. These measurements enabled us to figure out that the 2D passivation effectively passivates perovskite surface defects which leads to reduced non-radiative interfacial recombination.

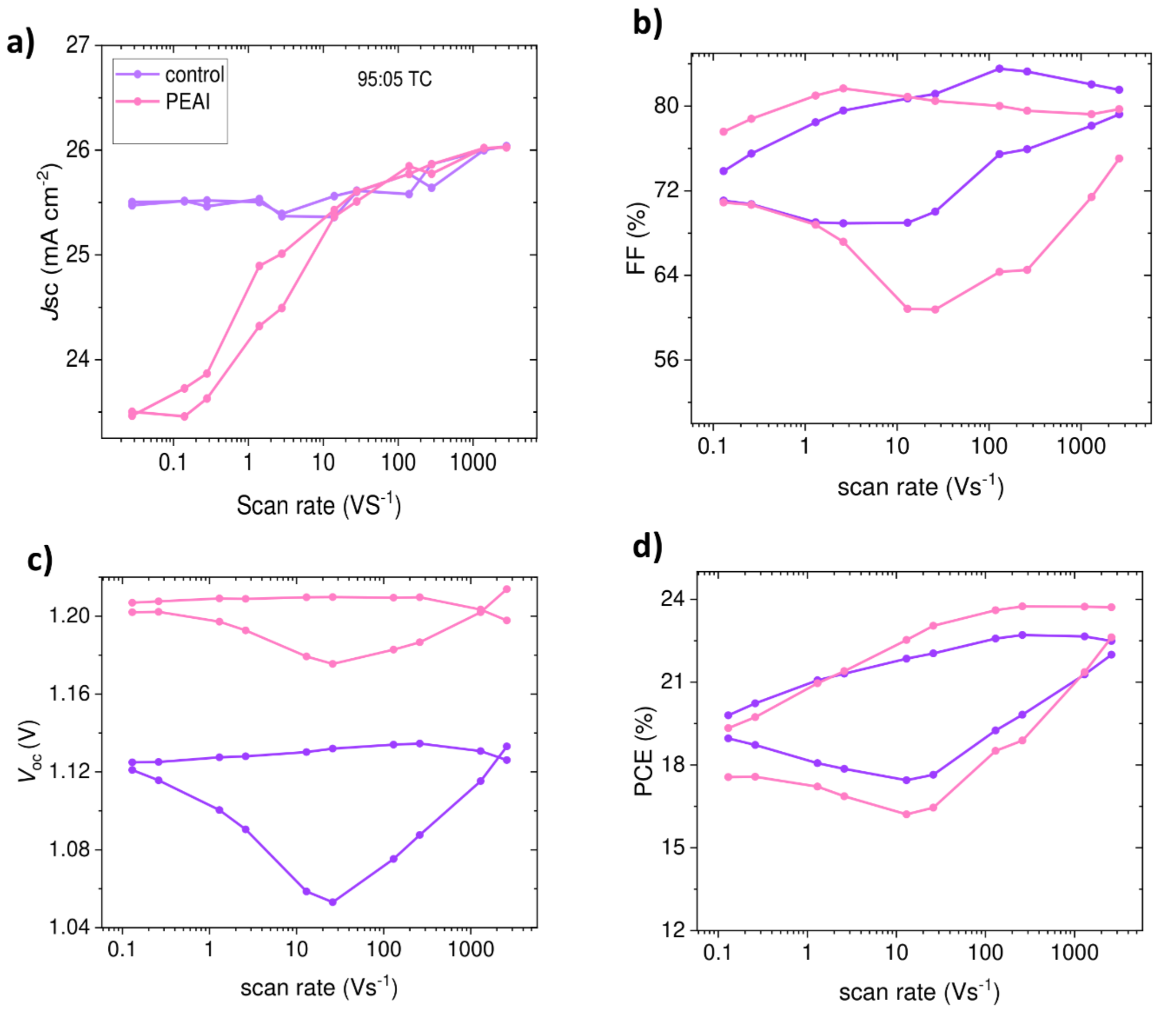

Figure S5: A direct comparison of J_{SC} (a), FF (b), V_{OC} (c), and PCE (d) from Fast hysteresis (FH) measurements of non-passivated and PEAI-passivated perovskite devices which show large ionic J_{SC} loss in PEAI-passivated devices

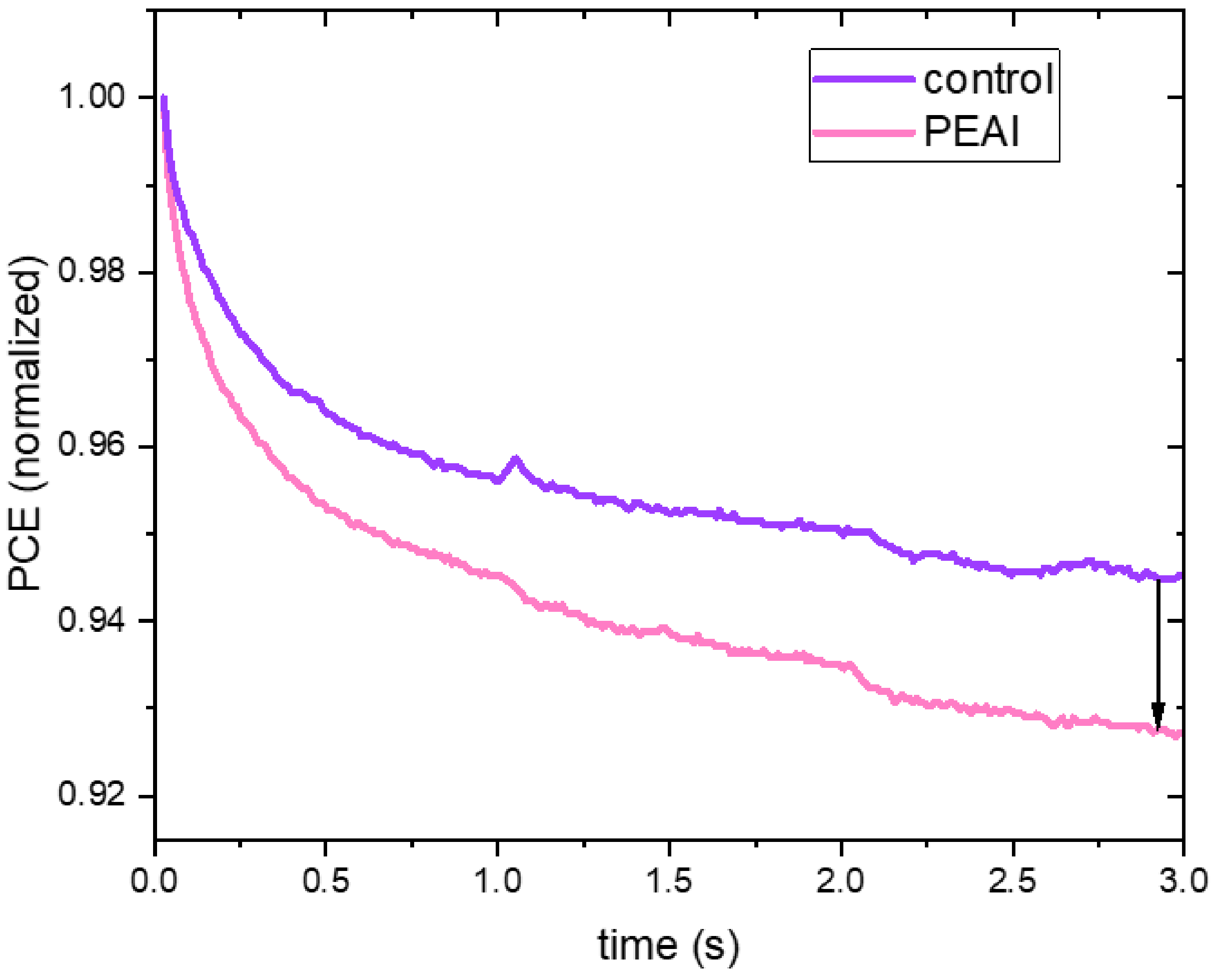

Figure S6: The efficiency decay on short timescales as obtained through maximum power point measurements. This figure highlights the MPP tracking of control and PEAI-passivated devices which was taken at the very beginning of the measurement. The passivated device exhibits a rapid initial decline in maximum power output, a phenomenon that could be easily overlooked when evaluating device efficiency over extended time periods. The formation of field-free regions within the perovskite bulk, caused by mobile ions, is not only a key factor contributing to *JV* hysteresis but also leads to substantial current losses and efficiency degradation under real-world operating conditions.

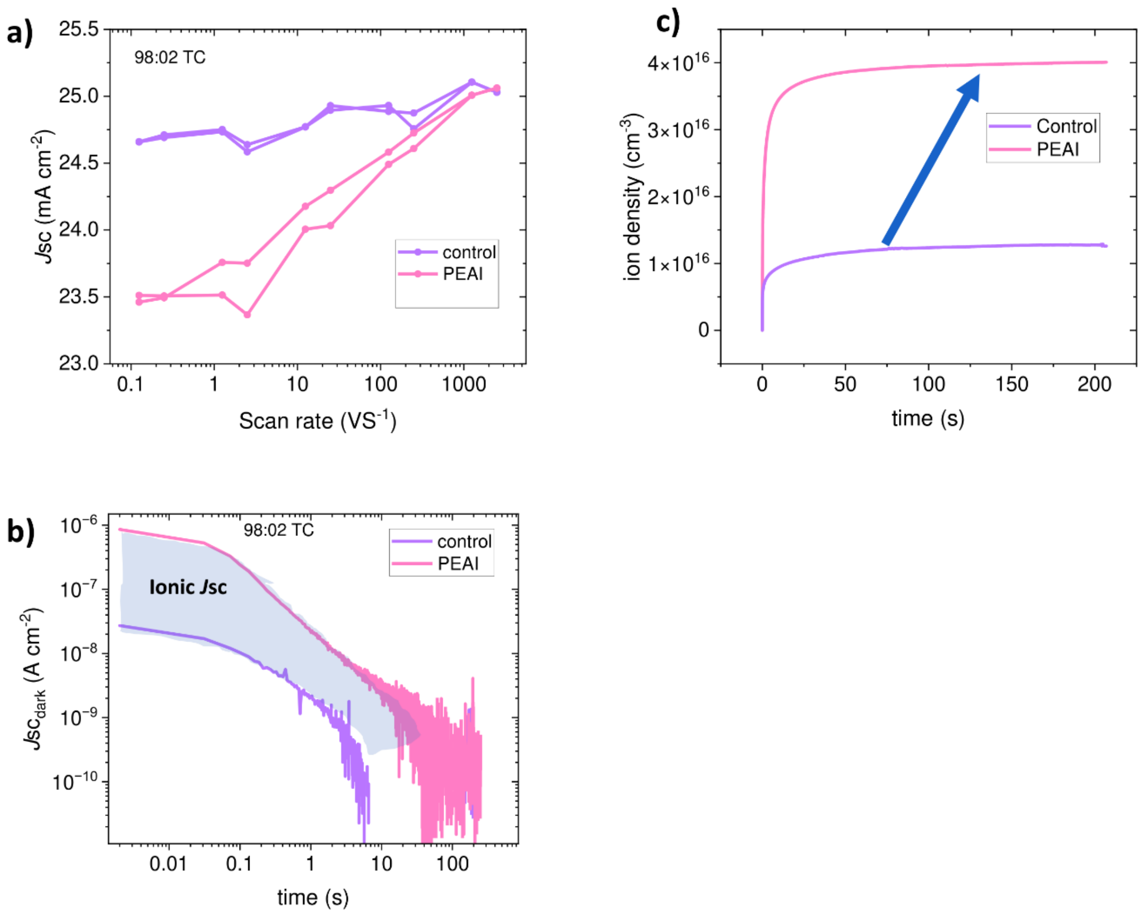

Figure S7: a) J_{SC} from FH measurements measured at different scan speeds for control and passivated device on the 98:2 TH perovskite composition. b), c), Current transients from BACE measurements for both control and passivated devices (a) and integrated ion density from the transients in (c).

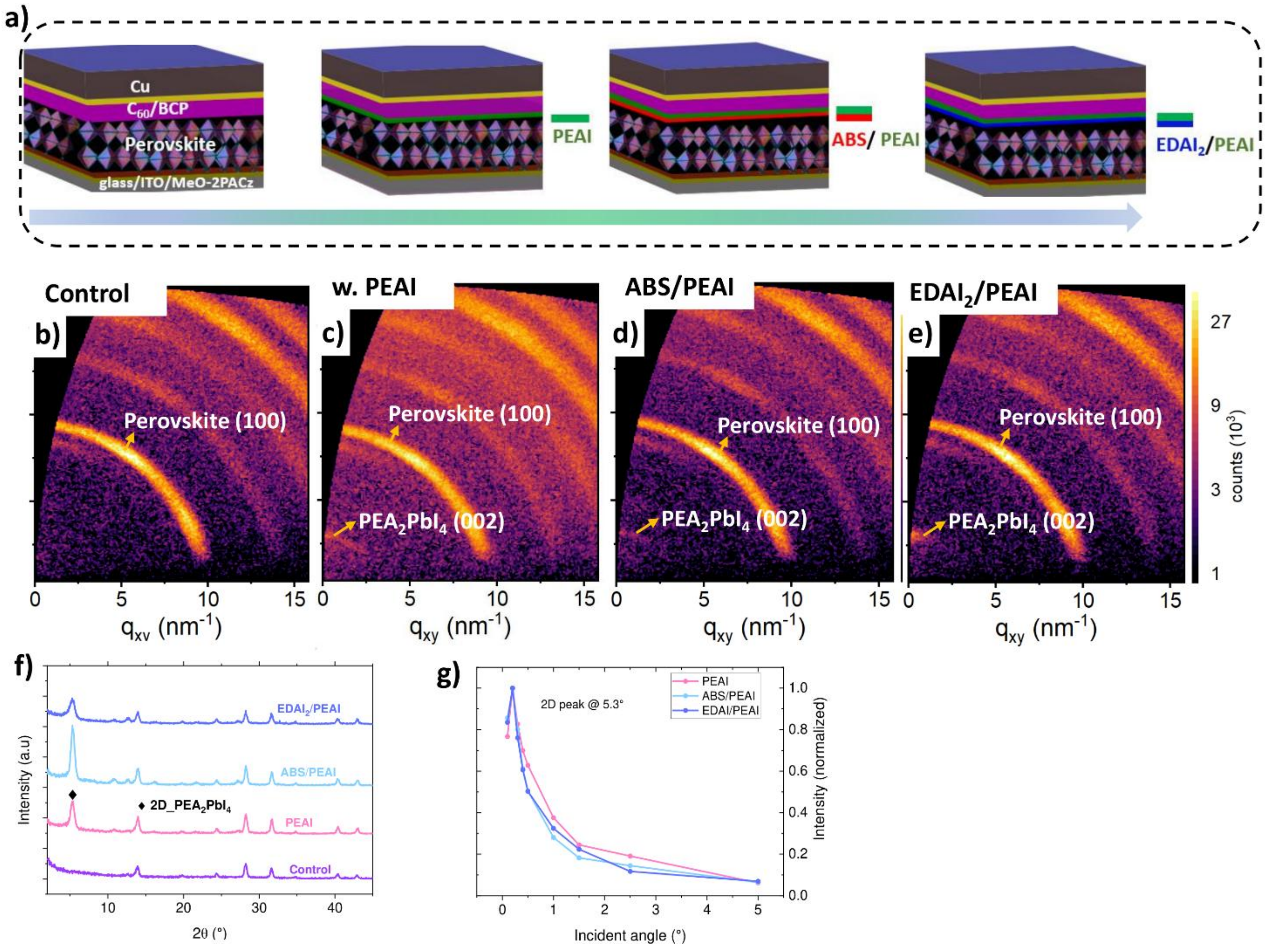

Figure S8. a) The full device scheme shows control and the three combinations of 2D/3D perovskites investigated in this work. (b-e) The GIWAXS images of control, PEAI, ABS/PEAI, and $EDAI_2$/PEAI-based devices taken at an incident angle of 3°. f) The corresponding GIXRD patterns and g) The normalized GIXRD peak intensity of the 2D PEA_2PbI_4 as a function of different incidence angles.

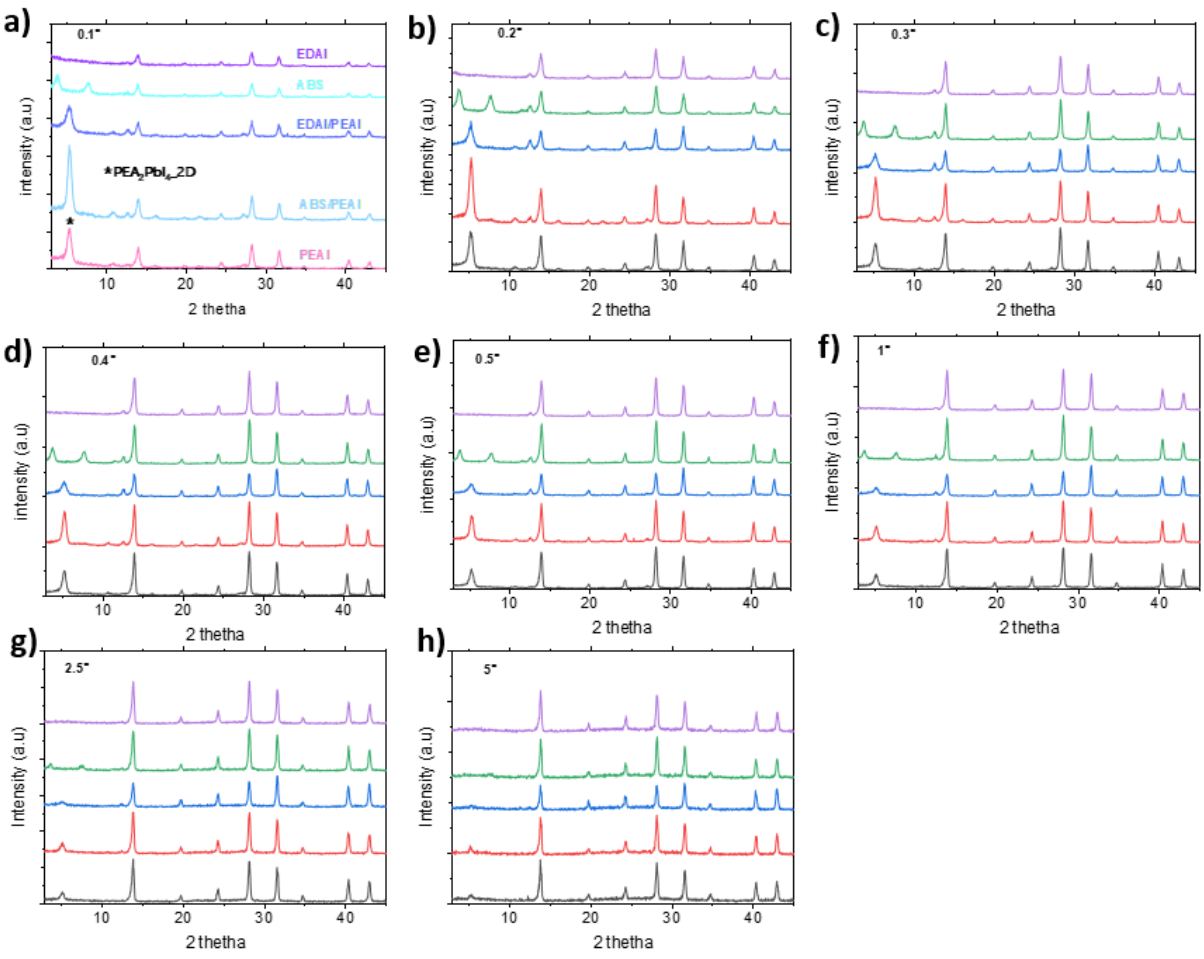

Figure S9. f) The GIXRD patterns of control, PEAI, ABS, $EDAI_2$, ABS/PEAI, and $EDAI_2$/PEAI-based devices at different angles on incident (0.1°, 0.2°,0.3°, 0.4°, 0.5°, 1°, 2.5, and 5°).

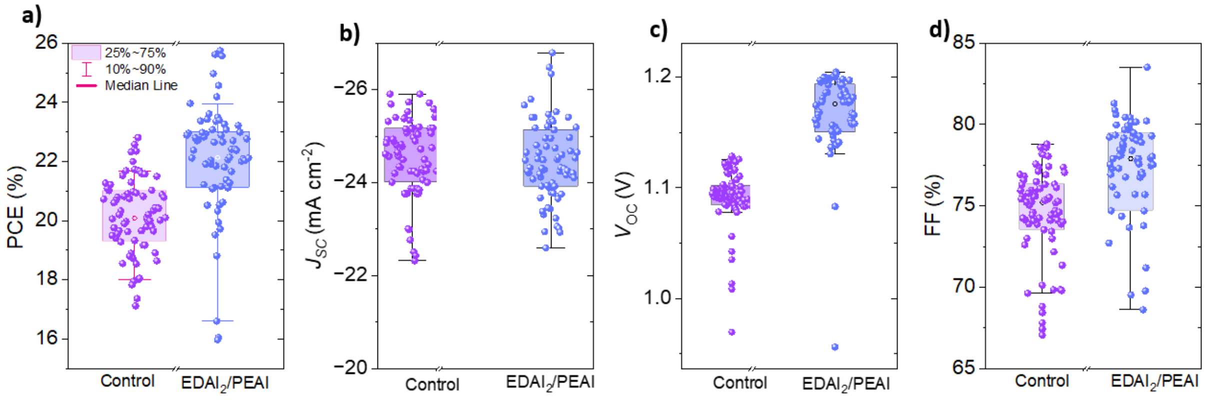

Figure S10. a, b, c, d) Statistics of comparison of PV parameters of control and $EDAI_2$/PEAI-passivated devices taken from 15 individual solar cells and active area of 6 mm^2: a) power conversion efficiency (PCE) b) short circuit current density (J_{SC}) c) open circuit voltage (Voc), and d) fill factor (FF),

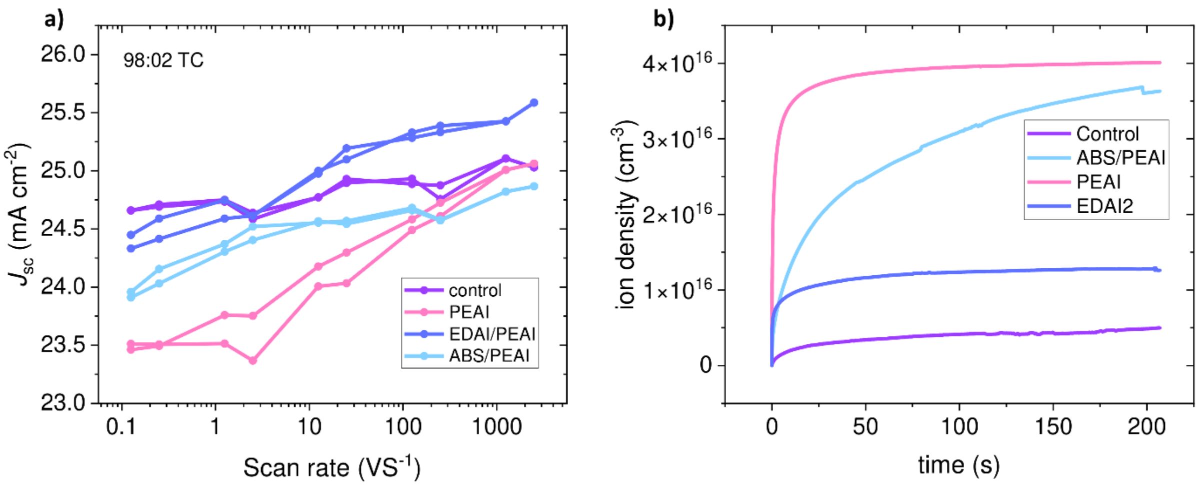

Figure S11: a) J_{SC} from FH measurements measured at different scan speeds for control, PEAI, ABS/PEAI, and $EDAI_2$/PEAI and passivated device on the 98:2 TH perovskite composition. b) integrated charge density obtained from the BACE measurements for control, PEAI, ABS/PEAI, and $EDAI_2$/PEAI-based devices. Whilst the cell with a PEAI-only passivation layer shows high ionic J_{SC} loss, the $EDAI_2$ and or ABS-containing device displays a slight drop in J_{SC} which is likely due to the reduced ion density in this sample, a similar phenomenon observed in 95:05 TH perovskite composition.

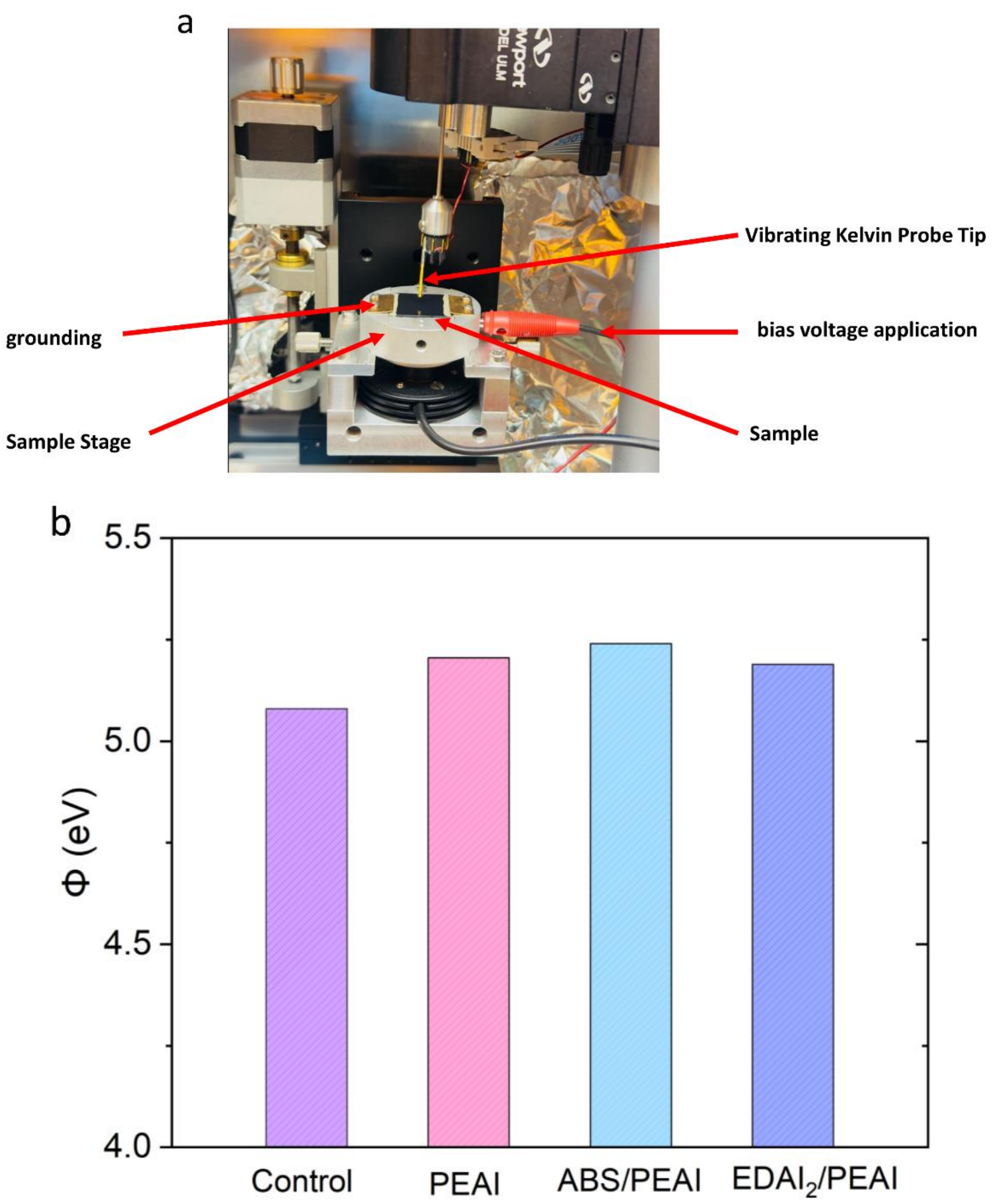

Figure S12: (a) Kelvin Probe measurement setup used for work function characterization. The system includes a vibrating Kelvin probe tip positioned above the sample stage, with electrical connections for grounding and bias voltage application. (b) Measured work function (Φ) values for control and passivated samples.

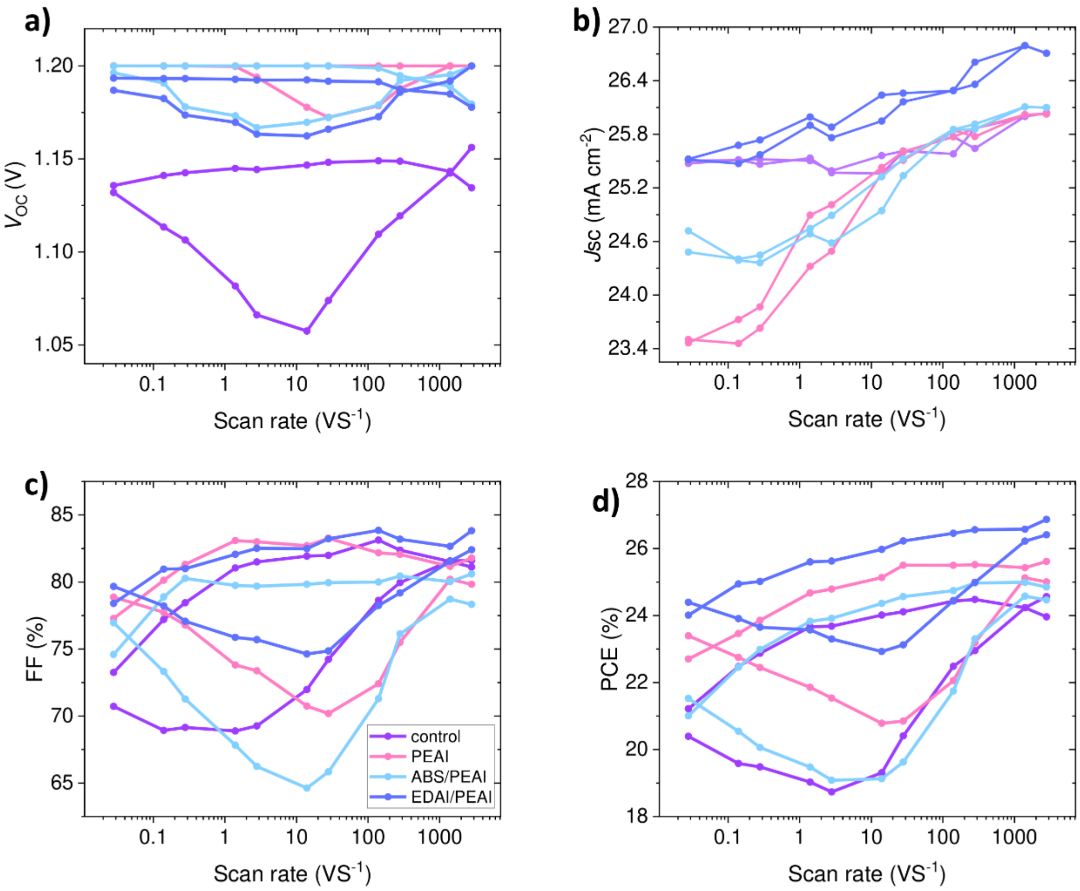

Figure S13: a) *PV* parameters obtained from FH measurements measured for control, PEAI, ABS/PEAI, and $EDAI_2$/PEAI passivated devices. V_{OC} (a), J_{SC} (b), FF (c), and PCE (d). The FF and V_{OC} did not show scan rate-dependent loss irrespective of the different passivation types, what most affected was the $J_{SC.}$

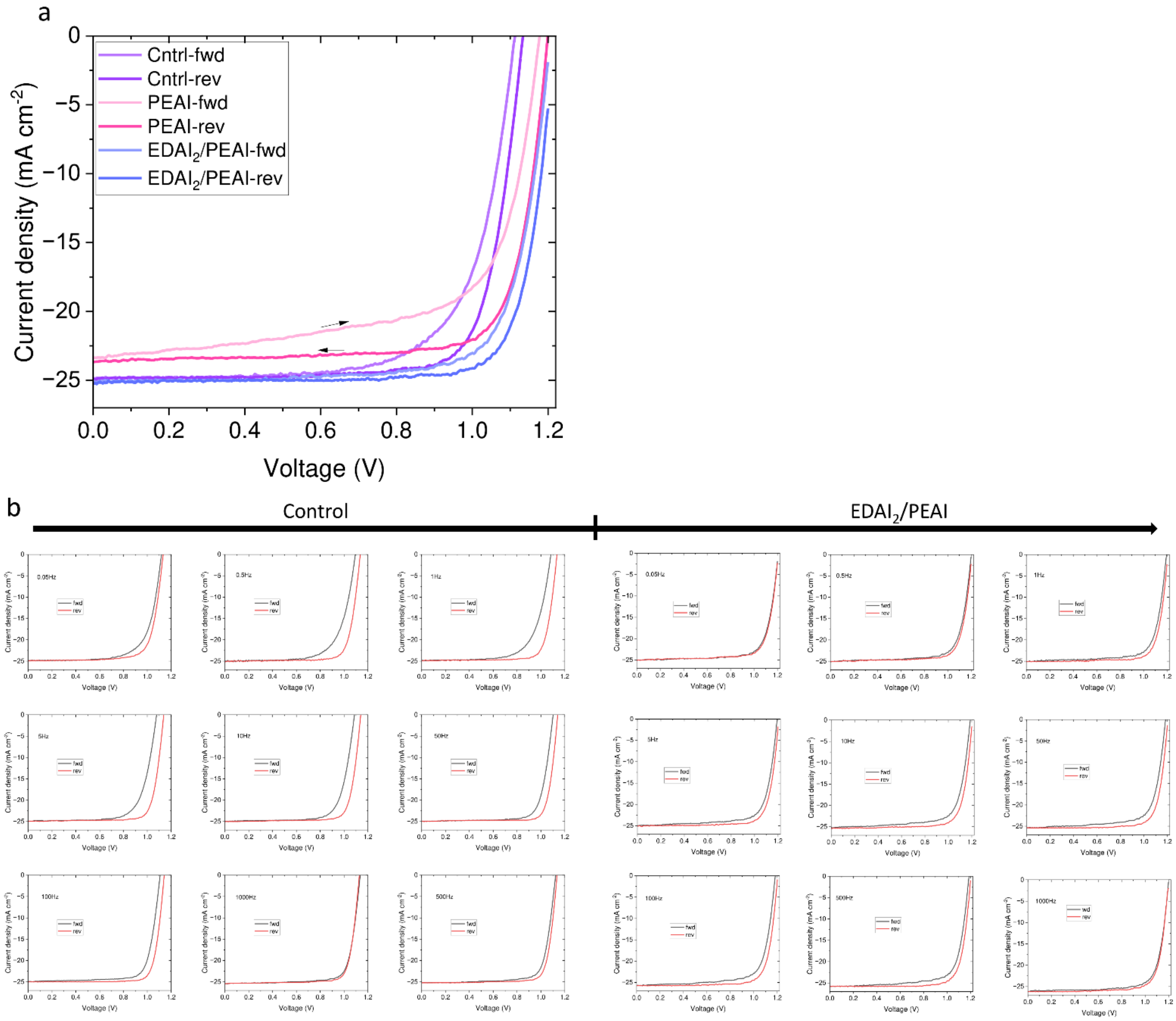

Figure S14. *J-V* characteristics of control, PEAI-passivated, and $EDAI_2$/PEAI bilayer-passivated perovskite solar cells measured in both forward (Fwd) and reverse (Rev) scan directions. The PEAI-only device exhibits noticeable hysteresis, indicating mobile ion migration. In contrast, the $EDAI_2$/PEAI bilayer passivation significantly reduces hysteresis, suggesting improved charge extraction and suppressed ion migration.

PEAI-passivated devices exhibit noticeable hysteresis, indicating mobile ion redistribution, while $EDAI_2$/PEAI bilayer-passivated devices show reduced hysteresis, suggesting improved charge extraction and ion suppression. This aligns with our fast hysteresis (FH) measurements, confirming that hysteresis in PEAI devices is driven by mobile ion accumulation, which our bilayer strategy effectively mitigates.

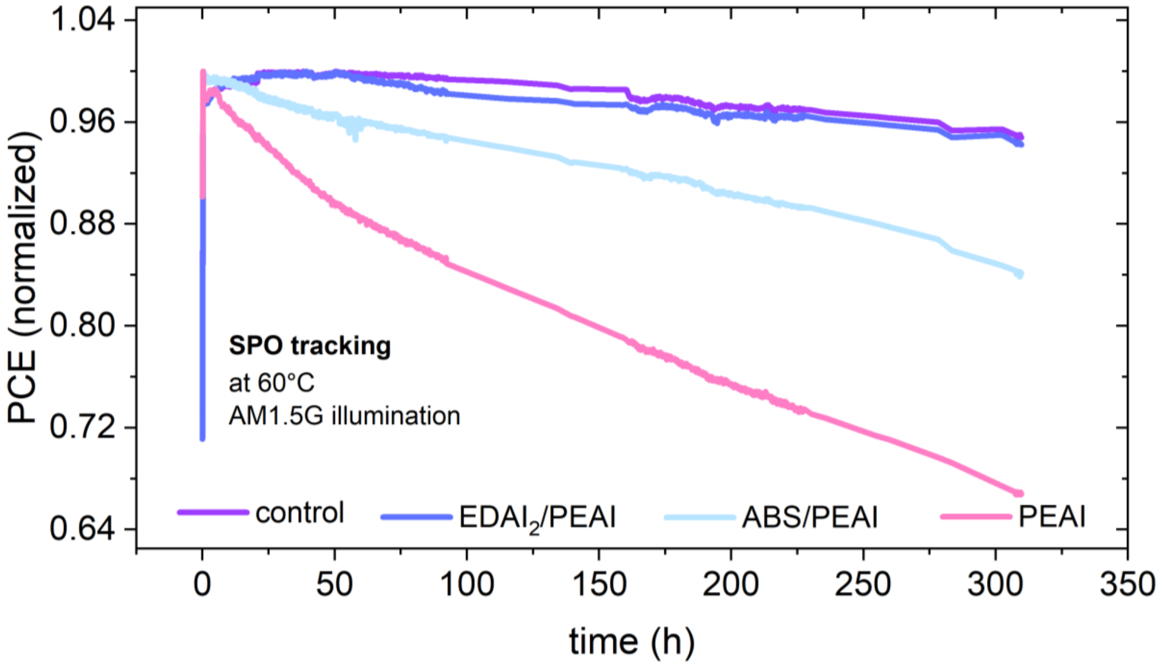

Figure S15. Maximum power point tracking (MPPT) stability of control, PEAI-passivated, EDAI$_2$/PEAI, and ABS/PEAI bilayer-passivated perovskite solar cells at 60°C. The PEAI-only device exhibits severe degradation over time, highlighting the impact of mobile ion migration on device instability. In contrast, bilayer-passivated devices (EDAI$_2$/PEAI and ABS/PEAI) demonstrate significantly improved stability, maintaining a higher fraction of their initial performance. These results confirm the effectiveness of bilayer passivation in mitigating thermal degradation and ensuring long-term operational stability.

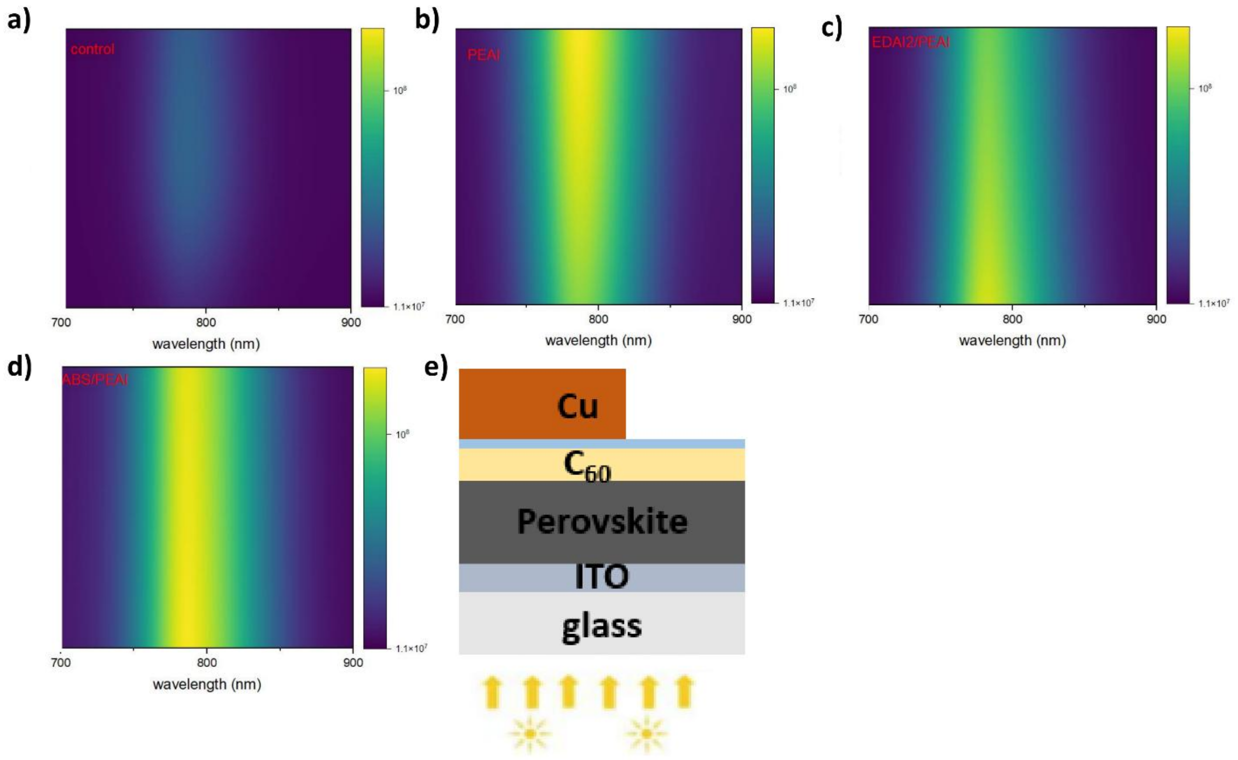

Figure S16: a-d) Shows the time dependency of the evolution of the photoluminescence spectra during continuous illumination with 1 Sun (a) control (b) PEAI passivated (c) $EDAI_2$/PEAI, and (d) ABS/PEAI passivation devices. e) Schematic drawing of the device stack. It has to be noted, that the PL peak position of all devices remains unchanged and remains stable with continuous illumination, with the position of the peak centered at ∼780 nm (1.58 eV) which is consistent with the bandgap calculated from EQE, highlighting that there is no phase segregation even in 2D passivated devices. The high PL emission intensity in passivated devices is due to surface passivation which passivates the defect within the perovskite surface or in the bulk. This figure displays the photoluminescence (PL) of the full stack after continuous illumination, following our previous method.[16] Specifically, the devices were illuminated under AM 1.5G conditions for 45 seconds, and the evolution of the PL spectrum was tracked over time. The constant PL peak position indicates no phase segregation, regardless of the different 2D passivation layers used. Additionally, the increased PL intensity demonstrates the effective passivation by the various 2D materials, enhancing emission and corroborating the improved photoluminescence quantum yield (PLQY).

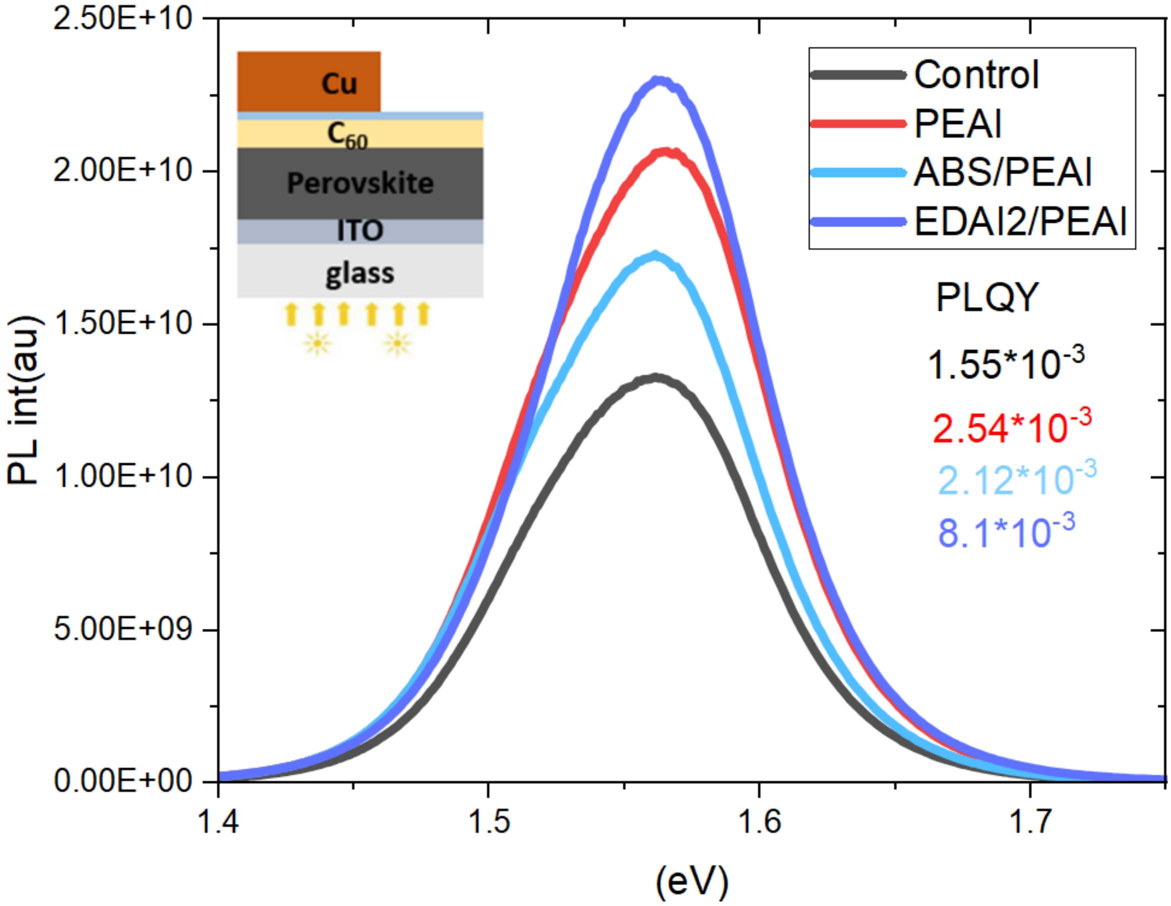

Figure S17: Photoluminescence quantum yield (PLQY) spectra of full solar cell stacks for control, PEAI, ABS/PEAI, and EDAI$_2$/PEAI-based devices at 520 nm excitation wavelength, highlighting a 2D-induced improvement in the PL emission and PLQY, mainly in a device treated with EDAI$_2$/PEAI bilayer, which is in agreement with the high V_{OC} in this system.

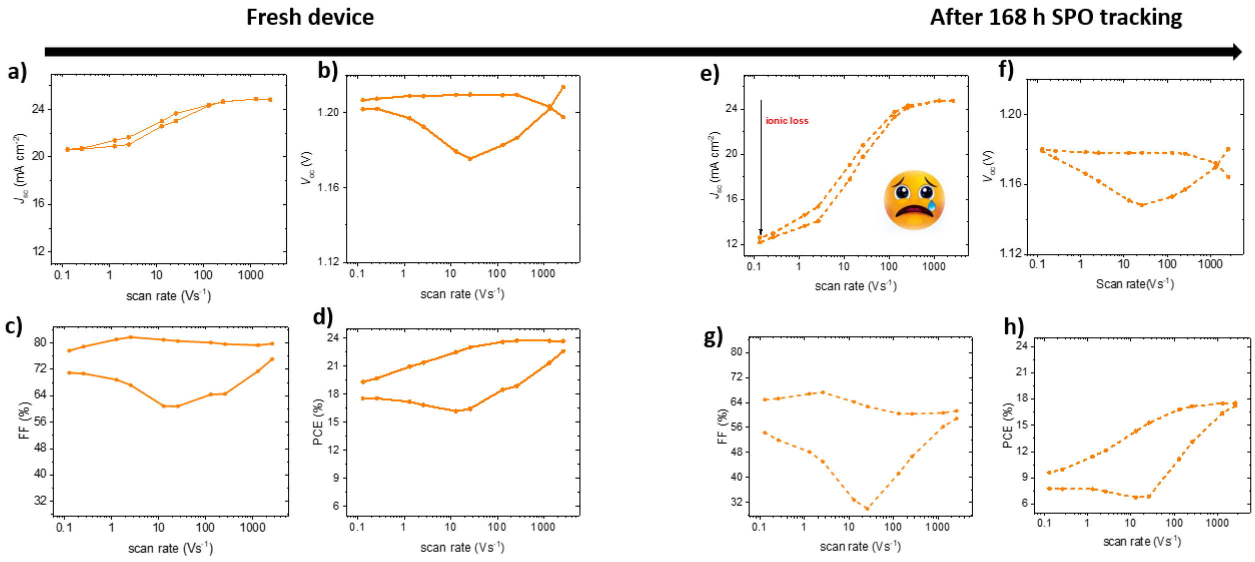

Figure S18: a) The short-circuit current density (J_{SC}), b) open-circuit voltage (VOC), c) fill factor (FF), and d) power conversion efficiency (PCE) obtained from Fast hysteresis (FH) measurements of PEAI-passivated device before SPP tracking. e) the short-circuit current density (J_{SC}), f) open-circuit voltage (VOC), g) fill factor (FF), and h) power conversion efficiency (PCE) obtained from Fast hysteresis (FH) measurements of PEAI-passivated device after 168 h SPP tracking.

References:

1. Seid, B. A. *et al.* Understanding and Mitigating Atomic Oxygen-Induced Degradation of Perovskite Solar Cells for Near-Earth Space Applications. *Small* **2311097**, 1–10 (2024).
2. Li, Z. *et al.* Organometallic-functionalized interfaces for highly efficient inverted perovskite solar cells. *Science* **376**, 416–420 (2022).
3. Lang, F. *et al.* Influence of Radiation on the Properties and the Stability of Hybrid Perovskites. *Adv. Mater.* **30**, 1702905 (2018).
4. Thiesbrummel, J. *et al.* Ion-induced field screening as a dominant factor in perovskite solar cell operational stability. *Nat. Energy* **9**, 664–676 (2024).
5. Advanced Energy Materials - 2020 - Kirchartz - Photoluminescence-Based Characterization of Halide Perovskites for.pdf.
6. Lucarelli, G., De Rossi, F., Taheri, B., Brown, T. M. & Brunetti, F. Phenethylammonium Iodide Passivation Layers for Flexible Planar Perovskite Solar Cells. *Energy Technol.* **10**, 202200314 (2022).
7. Herterich, J. *et al.* Toward Understanding the Short-Circuit Current Loss in Perovskite Solar Cells with 2D Passivation Layers. *Sol. RRL* **6**, 202200195 (2022).
8. Sutanto, A. A. *et al.* In Situ Analysis Reveals the Role of 2D Perovskite in Preventing Thermal-Induced Degradation in 2D/3D Perovskite Interfaces. *Nano Lett.* **20**, 3992–3998 (2020).
9. Liu, T. *et al.* Tuning interfacial energetics with surface ligands to enhance perovskite solar cell performance. *Cell Reports Phys. Sci.* **4**, 101650 (2023).
10. Li, J. *et al.* Universal Bottom Contact Modification with Diverse 2D Spacers for High-Performance Inverted Perovskite Solar Cells. *Adv. Funct. Mater.* **31**, 202104036 (2021).
11. Perini, C. A. R. *et al.* Interface Reconstruction from Ruddlesden–Popper Structures Impacts Stability in Lead Halide Perovskite Solar Cells. *Adv. Mater.* **34**, 202204726 (2022).
12. Zhu, H. *et al.* Tailored Amphiphilic Molecular Mitigators for Stable Perovskite Solar Cells with 23.5% Efficiency. *Adv. Mater.* **32**, 201907757 (2020).
13. Chen, J., Lee, D. & Park, N. G. Stabilizing the Ag Electrode and Reducing J-V Hysteresis through Suppression of Iodide Migration in Perovskite Solar Cells. *ACS Appl. Mater. Interfaces* **9**, 36338–36349 (2017).
14. Chen, P. *et al.* In Situ Growth of 2D Perovskite Capping Layer for Stable and Efficient Perovskite Solar Cells. *Adv. Funct. Mater.* **28**, 201706923 (2018).

15. Huang, Y. *et al.* Finite perovskite hierarchical structures via ligand confinement leading to efficient inverted perovskite solar cells. *Energy Environ. Sci.* **16**, 557–564 (2022).

16. Peña-Camargo, F. *et al.* Halide Segregation versus Interfacial Recombination in Bromide-Rich Wide-Gap Perovskite Solar Cells. *ACS Energy Lett.* **5**, 2728–2736 (2020).
